# Computational analysis of coiled tubing concerns during oil well intervention in the upper Assam basin, India

**DOI:** 10.1038/s41598-022-26670-5

**Published:** 2023-01-31

**Authors:** Dhrubajyoti Neog, Ankur Sarmah, Mohammad Irfan Sunny Baruah

**Affiliations:** Department of Petroleum Technology, Dibrugarh University, Dibrugarh, 786004 India

**Keywords:** Energy science and technology, Engineering

## Abstract

Coil tubing (CT) is widely regarded as one of the most effective servicing tools for dealing with a variety of oil and gas production issues, and it is also commonly used for oil well workover operations in India's upper Assam basin. The current work considers QT 800 to be the CT material used for actual oil well operations. With reference to actual operations carried out in some of the oil wells in the upper Assam basin, the current research analyses the limitations of CTs (QT 800, QT 900, and QT 1000) based on developing limit curves that can depict the operating limit and infer CT failure probability. This study also includes fatigue analysis to determine the likelihood of damage from hot oil circulation, water injection, and nitrogen shot operations while performing them using the CTs (QT 700, QT 800, QT 900, and QT 1000). The current work adopts the methodology of CT assessment based on a computational model built in MATLAB with respect to different oil well parameters in the upper Assam basin. This study takes an innovative approach by taking downhole temperature into account when determining the CT limit for QT 800, which signifies novelty in the current work. According to the computational analysis used in the study on CT limits, mechanical strain, thermal strain, and the combined strain of the CT material all affect CT elongation. This observation was often found to be a research gap in different research works as this aspect in previous studies was not considered while analysing CT operations. The findings of the present study highlight and draw the conclusion that temperature variations in the well and the CTU’s circulating fluid contribute linearly to CT strain. CT’s working limit diminishes with increasing internal and external pressure and diametrical growth, which eventually causes fatigue damage.

## Introduction

In recent years, the applications of coiled tubing have expanded to include drilling and completion activities as well as nitrogen kick-off, well cleaning, well stimulation (acidizing), well logging, cementing, and fishing operations^1–4^. Because CT is designed to operate without interfering with crude oil production, it can lower the cost of well service or maintenance, which is especially beneficial in high-pressure oil wells. In gas wells with high well head pressures, coil tubing can be employed. The CT outperforms typical snubbing devices in terms of cost savings and safety^5^. This increases well-productive time, which has an impact on the company’s long-term profitability. The primary advantage of CT in lowering operational time has piqued the oil industry’s interest, and it has established itself as a dependable, practical, and cost-effective option for oil well intervention activities. Coiled tubing, on the other hand, has limitations in terms of how long it can be used and how long it can operate. The operational constraints of CT are controlled by its ability to perform under high internal pressure and tension limits, life constraints due to fatigue and corrosion effects, and changes in the diameter and ovality limits^6^. Furthermore, when operating in a well with high temperatures and depths, the steel pipe experiences severe tensile stress and tension limit. The bending caused by the passage of coiled tubing on and off the reel and over the gooseneck results in plastic deformation of the pipe. This is an inescapable aspect of coiled tube operation, and it raises concerns about decreased CT yield^6^. CT yield criteria are subjected to axial (${{\varvec{\sigma}}}_{{\varvec{a}}}$), radial (${{\varvec{\sigma}}}_{{\varvec{r}}}$), and tangential (or hoop, ${{\varvec{\sigma}}}_{{\varvec{h}}}$) stresses, and the combined influence of these stresses is used to determine the material's yield. Numerous studies on the effects of stress on CTs have been conducted by various experts. Part of the coiled tubing is subjected to internal and external pressure due to fluid flow in the CT and the annulus between the CT and the production tubing. When the CT material is under tension or compression, axial forces act on it, causing axial stresses on the coiled tubing. When placing a CT in a well, the burst and collapse pressure ratings are considered. The burst pressure specification value provides vital information about the internal pressure that may damage or rupture the coiled tubing in the absence of external pressure and axial load. Similarly, in the absence of internal pressure and axial force, the collapse pressure can cause the CT walls to collapse. The Tresca and Von Mises yield criteria are commonly used to estimate the CT yield limit, which is a measure of the combined condition of all stresses. The Von Mises yield criterion was used in this work. This criterion demonstrates that yield occurs on the inner surface and that this should be taken into account when determining CT limits^6^.

The current work first discusses the different parameters to be included in the CT limit curve analysis and its fatigue estimation, and then displays a correlation to measure CT elongation with respect to different temperatures. Following this, the methodology for computational analysis of the coiled tubing materials, QT700, QT800, QT900, and QT1000, has been presented. The subsequent section presents the results of the findings in relation to oil well intervention operations carried out in actual well conditions. The current work reports the CT operational limits by considering the different sets of external and internal pressures, the addition of extra diametral growth (6%), and safety factor (SF) considerations. In this study, CT elongation was investigated with respect to actual nitrogen shooting and hot oil circulation (HOC) operations carried out using a QT 800 CT. The data used in the computational analysis relates to actual oil well operations by the CT in the upper Assam basin. The incorporation of the temperature effect is an innovative approach to studying the CT's performance, which most of the previous studies did not take into consideration. The present study finally draws a conclusion, highlighting the need to incorporate the novel effect of temperature on CT strain while analysing CT limitation. This finding signifies the novelty of the current work.

### CT limit curve

The limit curve behaviour was analysed in the current work to investigate the impact of the interrelationship of CT operating parameters, namely CT stresses, yield strength, and diameter increase. The CT is sensitive to internal and external pressure forces, including the axial force *F*_*a*_^6^. The CT limit curves were constructed by graphing different values of *F*_*a*_ and the accompanying pressure differential, (*∆P*), between internal (*P*_*i*_) and exterior pressures (*P*_*o*_) in a computational model in MATLAB to estimate its working limit in an oil well servicing environment. While the tube material is within the well, the limit curves assume *P*_*o*_ to remain constant. The limit curve shapes define the difference in the effects of the CT’s yield based on its external pressure. Furthermore, the diameter of the CT changes as a result of the fluid pressure influence on its body material. As a result, the limit curve with thin walls differs from the CT with thick walls. Apart from the diametral effect, CT bends as it is released from the reel, passes through the gooseneck, then coils back. The measurement of bending and internal pressure at the reel and guide arch aids in determining the distribution of fatigue damage along the string. It will also indicate when a pipe should be abandoned or when a portion of a CT pipe^7^ should be cut in order to maximise pipe utilisation while reducing pipe fatigue failure^8^. On the CT, bending causes ovality. In order to limit the extent of ovality creation, CT must be used with prudence. This is because bending a pipe deforms the cross-section and reduces the strength of the bend zone, causing buckling failure^9^. The guiding arch and reel have the highest bending stresses. The pipe may deform plastically if the stresses on the guiding arch and reel exceed the CT elastic yield strength^7^. Despite the fact that the internal pressure is smaller than the tubing’s yield strength, cyclic plastic bending and internal pressure cause CT diametral expansion and wall thinning^10^. CT suffers from low-cycle fatigue damage, and it occurs above the wellhead rather than in the well. Inside a well, CT remains in the elastic range^11^. CT is circulated while in a well, and it is lifted up after the desired circulation is attained. Because of repeated tripping actions, coiled tubing bends many times on the reel and gooseneck, resulting in plastic deformation and fatigue failure^12^. Fatigue damage and diametral expansion, on the other hand, can have different causes. A portion of the CT pipe fatigues owing to plastic deformation at the reel and guide arch despite the lack of internal pressure. Internal pressure, on the other hand, causes diametral expansion on CT. As a result, a string with a diameter increase will have considerable fatigue, while a string with no quantifiable diameter growth can not be presumed to have no fatigue damage^13^. When the tension and compressive forces caused by repeated bending exceed the yield strength of the CT, the working life is reduced. Furthermore, hoop stresses are induced by growing internal pressure in the CT, which enhances the rate of plastic deformation^14^. Table 1 shows the various equations and parameters involved in determining the limit curve in the current study with MATLAB.

**Table 1:** CT working limits^6^.

Parameters	Equation	Particulars
Axial Stress	$$\sigma_{a} = \frac{{F_{a} }}{A}\quad (1)$$	Tension mode; *A* = $$\pi ({{r}^{2}}_{o}$$*-*$${{r}^{2}}_{i })$$
	$\sigma_{a} = F_{a} \left[ {\frac{1}{A} + \frac{{Rr_{o} }}{2I}} \right]\quad (2)$	Compression mode
Radial Stress	${\sigma }_{r}=\frac{{{r}_{i}}^{2}{P}_{i- }{{r}_{o}}^{2 }{P}_{o}}{{{r}_{o}}^{2}-{{r}^{2}}_{i}}-\frac{\left({P}_{i}-{P}_{o}\right){{r}_{i}}^{2 }{{r}_{o}}^{2}}{\left({{r}_{o}}^{2} -{{r}_{i}}^{2}\right){r}^{2}}\quad (3)$	r is the radial location between ${r}_{i}\,and\, {r}_{o}$
Hoop Stress	${\sigma }_{h}=\frac{{{r}_{i}}^{2}{P}_{i- }{{r}_{o}}^{2 }{P}_{o}}{{{r}_{o}}^{2}-{{r}^{2}}_{i}}+\frac{\left({P}_{i}-{P}_{o}\right){{r}_{i}}^{2 }{{r}_{o}}^{2}}{\left({{r}_{o}}^{2} -{{r}_{i}}^{2}\right){r}^{2}}\quad (4)$	r is the radial location between ${r}_{i}\,and\, {r}_{o}$
Von Mises yield condition	$2{{\upsigma }_{\mathrm{y}}}^{2}= {\left({\upsigma }_{\mathrm{h}}-{\upsigma }_{\mathrm{r}}\right)}^{2}+{\left({\upsigma }_{\mathrm{h}}-{\upsigma }_{\mathrm{a}}\right)}^{2 }+{\left({\upsigma }_{\mathrm{a}}-{\upsigma }_{\mathrm{r}}\right)}^{2}\quad (5)$	CT yield mode
	${P}_{i}=\frac{-\gamma \pm \sqrt{{\gamma }^{2}-4\alpha \delta }}{2\alpha }\quad (6)$	Considers inner surface yield
	$\beta =\frac{{r}_{0}^{2}+{r}_{i}^{2}}{{r}_{0}^{2}-{r}_{i}^{2}}\quad (6a)$ $\alpha = {\beta }^{2}+\beta +1\quad (6b)$ $\gamma ={P}_{o}\left(2{\beta }^{2}+3\beta +1\right)+{\sigma }_{a}\left(\beta -1\right)\quad (6c)$ $\delta ={P}_{o}^{2}{\left(\beta +1\right)}^{2 }+{P}_{o}{\sigma }_{a}\left(\beta +1\right)+{\sigma }_{a}^{2}-{\sigma }_{y}^{2}\quad (6d)$	Defined parameter in Eq. (6)
CT wall thickness	${r}_{i2}=\sqrt{{{r}_{o2}}^{2}-2{r}_{o}w+{w}^{2}}\quad (7)$ ${w}_{2}={r}_{o2}-{r}_{i2}\quad (7a)$	Diameter growth

### CT fatigue damage

The CT is subjected to various stresses, plastic deformation, and internal pressure while in operation to restrict the fatigue life of the tube^15,16^. In addition, bending strains form on CT as a result of deployment and retrieval. In the absence of a straightener, strains are caused by the reel and the guiding arch. The addition of a straightener, on the other hand, results in reverse bending strain, which also has an effect on fatigue life, but it occurs in a minor elastic strain variation, and hence has a negligible effect^17^. An injector snubs CT in a high-pressure well against the wellhead pressure. In this case, the tubing within the injector and stripper is bent and unsupported. The supported zone fractures catastrophically as the compressive strain on the CT increases. The CT is bent by the gooseneck as it is drawn from the roller, through the gooseneck, and into the injector, and the chains inside the injector straighten the tubing as it goes through the injector. The material elastic energy causes the CT to rebound after being pushed by the injector. As a result, there is residual bending deformation in the CT. The gooseneck and rebounding curvature determine the residual curvature in the CT between the injector and the stripper. Straight and bending pipes have different stress distributions^18^. Furthermore, the service environment of CT causes mechanical damage to its outer surface. The outer surface of CT remains in contact with a rough and dynamic environment, which results in developing different forms of scratches, dents, and cracks on it. This type of mechanical damage often leads to the aggravation of fractures in the affected area and, subsequently, low cycle fatigue failure of coiled tubing^19^. Furthermore, material overload and material inadequacies are the two most typical causes of CT string failure^20^. Many studies find that the primary cause of CT failure is fatigue degradation caused by mechanical causes in addition to the corrosion effect on it^19,21^. Because CT is subjected to axial force, internal pressure, and bending strain while in operation, fatigue damage is unavoidable^21^. The routine examination of CTs following each well intervention may aid in the procedure involving the least amount of risk. Table 2 quantifies the bending stresses on CT induced by its bending on and off reel and gooseneck. The current study investigates the fatigue damage of various CT materials when used in oil wells for workover and servicing procedures, such as hot oil injection, water injection, and nitrogen shooting jobs. This study used the mathematical expressions in Table 2 to build the computational model in MATLAB to quantify CT fatigue. Several factors, including CT bending stresses, internal pressure, CT wall thickness and size, yield strength compensation factor, reel diameter and gooseneck radius, service conditions, and prediction reliability, were evaluated in the present work when calculating CT fatigue life consumed for a piece of tubing.

### CT elongation

The current study proposes a correlation to investigate CT elongation in oil field conditions. This analysis posits that when a CT pipe is inserted into an oil well, it elongates due to two factors: first, its own weight, which causes mechanical strain, and second, the varied temperature conditions to which it is exposed. When an extremely cold or very hot fluid is cycled through the CT, the pipe elongates or compresses according to the temperature differential, causing a degree of temperature strain. Based on the assumptions indicated above, the current study developed a correlation for measuring CT elongation (Eq. 22).

Temperature strain in the CT pipe,

20 $$ \begin{gathered} L_{f} = L_{i} \left( {1 + \alpha \Delta T} \right) \hfill \\ L_{f} = L_{i} + L_{i} \alpha \Delta T \hfill \\ \frac{{L_{f} - L_{i} }}{{L_{i} }} = \alpha \Delta T \hfill \\ \frac{\Delta L}{{L_{i} }}\left( t \right) = \alpha \Delta T \hfill \\ \end{gathered} $$

Mechanical strain in the CT pipe,

21 $$ \begin{gathered} \frac{\Delta L}{{L_{i} }}\left( m \right) = \frac{{\sigma_{a} }}{E} - \mu \frac{{\sigma_{h} }}{E} - \mu \frac{{\sigma_{r} }}{E} \hfill \\ \frac{\Delta L}{{L_{i} }}\left( m \right) = \frac{{F_{a} }}{EA} - \frac{\mu }{E}\left[ {\sigma_{h} + \sigma_{r} } \right] \hfill \\ \frac{\Delta L}{{L_{i} }}\left( m \right) = \frac{{F_{a} }}{EA} - \frac{\mu }{E}\left[ {2\frac{{r_{i}^{2} P_{i} - r_{0}^{2} P_{0} }}{{r_{0}^{2} - r_{i}^{2} }}} \right] \hfill \\ \frac{\Delta L}{{L_{i} }}\left( m \right) = \frac{{F_{a} }}{EA} - \frac{2\mu }{E}\left[ {\frac{{d_{i}^{2} P_{i} - d_{0}^{2} P_{0} }}{{d_{0}^{2} - d_{i}^{2} }}} \right] \hfill \\ \end{gathered} $$

Total strain in the CT pipe is given by adding the final Eqs. (20) and (21) of CT elongation due to temperature and axial load.

22 $$ \begin{gathered} \frac{\Delta L}{{L_{i} }} = \frac{\Delta L}{{L_{i} }}\left( m \right) + \frac{\Delta L}{{L_{i} }}\left( t \right) \hfill \\ \frac{\Delta L}{{L_{i} }} = \frac{{F_{a} }}{EA} - \frac{2\mu }{E}\left[ {\frac{{d_{i}^{2} P_{i} - d_{0}^{2} P_{0} }}{{d_{0}^{2} - d_{i}^{2} }}} \right] + \alpha \Delta T \hfill \\ \end{gathered} $$

wherein, $${\text{F}}_{{\text{a}}} = \left( {{\text{Weight}}\;{\text{per}}\;{\text{unit}}\;{\text{length}}\;{\text{of}}\;{\text{CT}}\;{\text{pipe}}\; \times \;{\text{Length}}\;{\text{of}}\;{\text{CT}}\;{\text{pipe}}} \right) + {\text{W}}_{{{\text{BHA}}}}$$

The above correlation does not include the stress concentration factor in the mathematical expression for CT elongation since it examines the temperature impact as a standalone mechanism to highlight its substantial significance in CT elongation.

## Methodology

Based on CT operations completed on a few oil wells in the upper Assam basin, India, the current study examines the working limits of three distinct coiled tubing materials, QT-800, 900, and QT-1000, with yield strengths of 80,000 psi, 90,000 psi, and 100,000 psi, respectively. The computational analysis initially examined the pressure and tension (P and T) limits of the CTs, with and without the effect of external pressure. The aforementioned CTs were then subjected to additional investigation into their P and T limitations, taking diametrical growth and the safety factor into account. The current work also included a fatigue analysis of coiled tubing materials (QT700, QT800, QT900, and QT1000) in relation to hot oil circulation, water injection, and nitrogen shot tasks. Following this, the mathematical correlation proposed for CT elongation was applied to examine CT strain as a function of reservoir temperature and axial load in a downhole context. In the CT elongation study, the hypothesised correlation was applied to a nitrogen shot operation and a hot oil circulation job on QT800. Because the QT 800 CT was used for a variety of operations in actual oil wells of upper Assam basin, the current study included it in the analysis of the temperature effect on CT elongation. The present study created a computational model to construct yield (P and T limit) curves and investigate fatigue damage in a variety of oil field well conditions. The results of the computational curves infer data related to CT operations on actual oil wells in the upper Assam basin. Finally, the current study analyses the working limit of CTs derived from computational analyses.

### MATLAB result validation

 The present study used a qualitative verification approach to validate MATLAB computational results, which limits the scope for comparison analysis. The computational results, however, are typical of model analyses of various CTU that are widely employed in oil field operations. In the current study, QT 800 is the CT with which some wells in the upper Assam basin were actually operated. CTU limits were investigated in relation to various well conditions in the current study; CTU test data were considered but not included in the comparative and quantitative analyses. Furthermore, the current study's goal is to develop limit curves that provide adequate data for evaluating CT damage caused by oil field operations, based on an intensive computational analysis of the CT problems using MATLAB.

This current work depicts a computational study which was conducted in MATLAB software using equations for limit curves referred to under sections “CT limit curve” in Table 1, “CT fatigue damage” in Table 2, and by using the proposed equation incorporated in section “CT Elongation” of Eq. 22. The CT models used in the study are QT 800, QT 900, and QT 1000 for pressure and tension limit studies. CT models used for CT fatigue damage were QTs at 700, 800, 900, and 1000. CT 800 was used for the CT elongation study. Table 3 under “Pressure and tension limits” section, Table 4 under “Pressure and tension limit curves for CTs at zero external pressure” section, Table 5 under “Pressure and tension limit curves for different external pressure” section, Table 6 under “Pressure and tension limit curves considering 6% diametric growth” section, Table 7 under “Pressure and tension limit curves considering safety factor (SF)” section, Table 8 under “Fatigue damage of QT 700, QT 800, QT900 and QT1000 observed for 1000 psi circulation pressure (Hot oil)” section, Table 9 under “Fatigue damage of QT 700, QT 800, QT900 and QT1000 observed for 2000 psi circulation pressure (hot oil)” section, Tables 10 and 11 under “Fatigue damage of QT 700, QT 800, QT900 and QT1000 observed for 2000 psi circulation pressure (hot oil)” section, Tables 12 and 13 under “Fatigue damage of QT 700, QT 800, QT900 and QT1000 observed for 2500 psi circulation pressure (water injection)” section, Tables 14 and 15 under “Fatigue damage of QT 700, QT 800, QT900 and QT1000 observed for 3000 psi circulation pressure (hot oil)” section, Tables 16 and 17 under “Fatigue damage of QT 700, QT 800, QT900 and QT1000 observed for 2000 psi nitrogen shooting job” section contain raw data for CT specifications. Raw data used to calculate CT elongation and temperature effect can be found in Tables 18 and 19 in section “For QT 800 nitrogen shooting”and Tables 20 and 21 in section “For QT 800 hot oil circulation”. The computational model can be accessed at https://drive.google.com/drive/folders/1H31QnKsTPBAjD5Ay6r3CCsn-jCQ2N3L0.

**Table 2:** Mathematical expressions for estimating CT fatigue Life^6^.

Parameters	Equation	Particulars
CT Bending Stress	${S}_{a,r}=\frac{{d}_{o} E}{{D}_{r}+{d}_{o}}\quad (8)$	On and off the reel
	${S}_{a,g}=\frac{{d}_{o} E}{2\left({R}_{g} +\frac{{d}_{o}}{2}\right)}\quad (9)$	On and off the gooseneck
Hoop Stress on OD	${S}_{h}=\frac{{{2d}^{2}}_{i}{P}_{i}}{{{d}^{2}}_{o}-{{d}_{i}}^{2}}\quad (10)$	Considers internal pressure
Combined Stress on CT	${S}_{r}={S}_{a,r}+{\left({S}_{h}\right)}^{1.895}\quad (11)$ (reel)	Avakov et al. 1993 fatigue model
	$${S}_{g}={S}_{a,g}+{\left({S}_{h}\right)}^{1.895}\quad (12)$$(gooseneck)	
No. of cycles during one tubing stroke	${N}_{1}=1{\left(\frac{{S}_{r}}{{S}_{m}}\right)}^{2}+2{\left(\frac{{S}_{g}}{{S}_{m}}\right)}^{2}\quad (13)$	As per Avakov et al., (1994)
Fatigue damage or consumed life per tubing stroke	${FD}_{1}=\frac{{N}_{1}}{{K}_{Q} {N}_{m}} * 100\%\quad (14)$	Considers reliability factor,${\mathrm{K}}_{\mathrm{Q}}$
Reliability factor,${K}_{Q}$	${K}_{Q}= {11.47}^{{\frac{\mathit{ln}Q}{\mathit{ln}0.5}}^{\frac{1}{15}-1}}\quad (15)$	
Fatigue damage in corrosive	${FD}_{2}=\frac{1}{{K}_{c}}\frac{{N}_{1}}{{K}_{Q} {N}_{m}} * 100\%\quad (16)$	Corrosion factors, K_c_ = 1.0 water or nitrogen K_c_ = 0.66 cement or acid K_c_ = 0.5 for H_2_S
Fatigue damage due to combined effect of corrosion and stress factor in one stroke of the tubing	${FD}_{3}=\frac{1}{{K}_{c}. {K}_{s}}\frac{{N}_{1}}{{K}_{Q} {N}_{m}} * 100\mathrm{\%}\quad (17)$	Stress correction factor, K_s_ = 1.0 no butt-weld section K_s_ = 0.9 bias-welded section K_s_ = 0.6 tapered bias-weld section K_s_ = 0.5 poorly dressed butt-weld connection K_s_ = 0.31undressed butt-weld connection
CT ultimate strength compensation factor	${K}_{us}={\left(\frac{\mathit{ln}\left(1-RA\right)}{\mathit{ln}\left(0.47\right)}\right)}^{2}\quad (18)$	wherein RA: reduction of area in fraction RA = 0.53 for QT-700 CT and equivalent RA = 0.57 for QT-800 CT and equivalent RA = 0.60 for QT-1000 CT and equivalent
Fatigue damage for one tubing stroke will be	${FD}_{1}=\frac{1}{{K}_{c}. {{K}_{s}K}_{us }}\frac{{N}_{1}}{{K}_{Q} {N}_{m}} * 100\mathrm{\%}\quad (19)$	Considering corrosion, stress and ultimate strength compensation factor

**Table 3:** CT specifications.

CT	Outer diameter(in.)	Wall Thickness(in.)	Inner diameter (in.)	Pipe body yield load (lb)
QT800	1.25	0.087	1.076	25,430
QT900	1.25	0.087	1.076	28,610
QT1000	1.25	0.087	1.076	31,790

**Table 4:** P&T limit data for zero external pressure, *P*_*o*_.

CT Name	Axial load, ${\mathbf{F}}_{\mathbf{a}}$ (lb)	Internal Pressure, Pi		∆P (psi)	
		Burst (psi)	Collapse (psi)	Burst	Collapse
QT800	10,000	11,956.67	(-) 8555.45	11,956.67	(-) 8555.45
QT900	10,000	13,419.16	(-) 10,017.9	13,419.16	(-)10,017.9
QT1000	10,000	14,862.79	(–) 11,461.6	14,862.79	(-)1461.6

**Table 5:** Comparison of Limit curve data for different external pressures, *P*_*o*_.

CT Name	Axial Load Fa (lb)	Internal Pressure, Pi (psi)		ΔP Burst with external pressures (psi)			ΔP Collapse with external pressures (psi)		
		Burst	Collapse	0	2500	5000	0	2500	5000
QT800	25,000	8965.803	** − **462.754	8965.803			** − **462.754		
	25,000	10,867.06	2906.382		8367.064			406.3815	
	25,000	12,565.28	6478.558			7565.284			1478.558
QT900	25,000	11,624.59	** − **3121.54	11,624.59			** − **3121.54		
	25,000	13,813.54	** − **40.0976		11,313.54			** − **2540.1	
	25,000	15,956.19	3087.655			10,956.19			** − **1912.35
QT1000	25,000	13,753.14	** − **5250.1	13,753.14			** − **5250.1		
	25,000	16,046.39	** − **2272.94		13,546.39			** − **4772.94	
	25,000	18,315.01	728.8321			13,315.01			** − **4271.17
QT800	10,000	14,463.7	** − **5792.09		11,963.7			** − **8292.09	
QT900	10,000	15,942.36	** − **7270.74		13,442.36			** − **9770.74	
QT1000	10,000	17,398.37	** − **8726.76		14,898.37			** − **11,226.8	
QT800	10,000	16,959.1	** − **3017.09			11,959.1			** − **8017.09
QT900	10,000	18,455.77	** − **4513.76			13,455.77			** − **9513.76
QT1000	10,000	19,925.48	** − **5983.47			14,925.48			** − **10,983.5

**Table 6:** ΔP Limits for a 6% diameter growth.

P & T limit behaviour without any expansion or increase of CTs diameter									
CT Name (QT)	Axial Load, Fa(lb)	ΔP (burst) without diameter growth			ΔP (collapse) without diameter growth			Internal pressure, Pi	
		0 (psi)	2500 (psi)	5000 (psi)	0 (psi)	2500 (psi)	5000 (psi)	Burst	Collapse
800	10,000	11,956.67			** − **8555.45			11,956.67	** − **8555.45
			11,963.7			** − **8292.09		14,463.7	** − **5792.09
				11,959.1			8017.09	16,959.1	** − **3017.09
900	10,000	13,419.16			** − **10,017.9			13,419.16	** − **10,017.9
			13,442.36			** − **9770.74		15,942.36	** − **7270.74
				13,455.77			** − **9513.76	18,455.77	** − **4513.76
1000	10,000	14,862.79			** − **11,461.6			14,862.79	** − **11,461.6
			14,898.37			** − **11,226.8		17,398.37	** − **8726.76
				14,925.48			** − **10,983.5	19,925.48	** − **5983.47

P & T limit behaviour considering 6% expansion or increase of CTs diameter									
CT Name (QT)	Axial Load, Fa	ΔP (burst) considering diameter growth			ΔP (collapse) considering diameter growth			Internal pressure, Pi	
		0 psi	2500 psi	5000 psi	0 psi	2500 psi	5000 psi	Burst	Collapse
800	10,000	10,634.27			** − **7525.6			10,634.27	** − **7525.6
			10,645.7			** − **7290.12		13,145.7	** − **4790.12
				10,645.7			** − **7290.12	13,145.7	** − **4790.12
900	10,000	11,927.2			** − **8818.53			11,927.2	8818.53
			11,952.73			** − **8597.16		14,452.73	** − **6097.16
				11,969.74			8367.25	16,969.74	** − **3367.25
1000	10,000	13,203.64			** − **10,095			13,203.64	** − **10,095
			13,239.99			** − **9884.42		15,739.99	** − **7384.42
				13,268.95			** − **9666.46	18,268.95	** − **4666.46

**Table 7:** ΔP with or without a safety factor for burst and collapse pressure.

CT Name	∆P (burst) without SF (psi)			∆P (burst) considering SF (psi)			∆P (collapse) without SF (psi)			∆P (collapse) considering SF (psi)		
	0 psi	2500	5000	0	2500	5000	0psi	2500	5000	0psi	2500	5000
QT 800	11,956.67	11,963.7	11,959.1	9565.334	9570.964	9567.283	** − **8555.45	** − **8292.09	** − **8017.09	** − **4277.72	** − **4146.04	** − **4008.55
QT900	13,419.16	13,442.36	13,455.77	10,735.33	10,753.89	10,764.62	** − **10,017.9	** − **9770.74	** − **9513.76	** − **5008.97	** − **4885.37	** − **4756.88
QT 1000	14,862.79	14,898.37	14,925.48	11,890.23	11,918.7	11,940.39	** − **11,461.6	** − **11,226.8	** − **10,983.5	** − **5730.79	** − **5613.38	** − **5491.73

**Table 8:** Information on HOC job with CT.

Particulars	Data
Length	10,000 feet
Outer diameter	2 inches
Wall thickness	0.188 inch
Internal pressure	1000 psi
Welding location	3000, 6000,9000 feet
Reliability factor	0.95
Corrosion factor	1
Weld reduction factor	0.9 (Bias Welding)
Goose neck radius	72 inches
Reel radius	52 inches
Young’s Modulus	30,000,000 psi

**Table 9:** Comparison of fatigue damage for different CT material.

CT	Fatigue in a normal section (%)	Fatigue in a welded section (%)
QT700	1.72	2.18
QT800	1.38	1.7
QT900	1.27	1.58
QT1000	1.19	1.5

**Table 10:** Information on HOC job with CT (2000 psi).

Particulars	Data
Length	5500 feet
Outer diameter	1.25 inch
Wall thickness	0.087 inch
Internal pressure	2000 psi
Welding location	3000 feet
Reliability factor	0.95
Corrosion factor	1
Weld reduction factor	0.9
Goose neck radius	72 inches
Reel radius	72 inches
Young’s Modulus	30,000,000 psi

**Table 11:** Fatigue damage comparison for CTs in relation to HOC job at 2000 psi circulation pressure.

Material	Fatigue in non-welded parts (%)	Fatigue in welded parts (%)
QT700	7.6	9.5
QT800	6.1	7.6
QT900	5.6	7
QT1000	5.3	6.6

**Table 12:** Information on water injection project with CT.

Particulars	Data
Length	11,761 feet
Outer diameter	1.25 inch
Wall thickness	0.087 inch
Internal pressure	2500 psi
Welding location	3000, 6000,9000 feet
Reliability factor	0.95
Corrosion factor	1
Weld reduction factor	0.9
Goose neck radius	72 inches
Reel radius	72 inches
Young’s Modulus	30,000,000 psi

**Table 13:** Comparison of fatigue damage for various CTs.

CT Name	Fatigue in non-welded parts (%)	Fatigue in welded parts (%)
QT700	13.4	16.8
QT800	10.8	13.3
QT900	9.9	12.2
QT1000	9.3	11.6

**Table 14:** Information on HOC job with CT (Circulation pressure: 3000 psi).

Particulars	Data
Length	2482 m (8364 feet)
Outer diameter	1.25 inch
Wall thickness	0.087 inch
Internal pressure	3000 psi
Welding location	3000, 6000 feet
Reliability factor	0.95
Corrosion factor	1
Weld reduction factor	0.9
Goose neck radius	72 inches
Reel radius	72 inches
Young’s Modulus	30,000,000 psi

**Table 15:** Comparison of fatigue damage for different CTs.

CT	Fatigue in non-welded parts (%)	Fatigue in welded parts (%)
QT700	21.8	27.2
QT800	17.5	21.7
QT900	16	20
QT1000	15.2	18.9

**Table 16:** Nitrogen shooting job data.

Particulars	Data
Length	1500–2540 m (5055–8560 feet)
Outer diameter	1.25 inch
Wall thickness	0.087 inch
Internal pressure	2000 psi
Welding location	3000, 6000 feet
Reliability factor	0.95
Corrosion factor	1
Weld reduction factor	0.9
Goose neck radius	72 inches
Reel radius	72 inches
Young’s Modulus	30,000,000 psi

**Table 17:** Fatigue damage from the nitrogen shooting job.

CT	Fatigue at non welded parts (%)	Fatigue in welded parts (%)
QT700	7.7	9.5
QT800	6.1	7.5
QT900	5.5	7
QT1000	5.3	6.5

**Table 18:** Information on a nitrogen shooting operation using CT.

Particulars	Data
Well temperature	90 °C
Nitrogen temperature	− 10 °C
Material	QT800
Co efficient of thermal expansion	1.05E-5
Poisson’s ratio	0.28
Young’s modulus	3.12E + 07
Outer diameter of pipe	1.25 inch
Thickness	0.087 inch
Weight per unit length of pipe	152.86 lbs./feet

**Table 19:** CT strain analysis for the QT800 during the nitrogen shot operation.

OD (do) (in)	ID (di) (in)	Ultimate Stress (*σ*_*u*_) (psi)	Internal Pressure (*Pi*) (psi)	Axial load (*F*_*a*_)(lbs)	Co efficient of thermal expansion (α)	Thermal strain	$\text{X} = \frac{{\text{F}}_{\text{a}}}{\text{EA}}$	Y = $\frac{{\text{d}}_{\text{i}}^{2}{P}_{i}-{\mathrm{d}}_{0}^{2}{P}_{0}}{{\mathrm{d}}_{0}^{2}-{\mathrm{d}}_{\mathrm{i}}^{2}}$	Z=(2μ/E)*Y	ΔL/L*100	ΔL/L*100(Thermal consideration)
1.25	1.076	9.80E + 04	3.00E + 03	0.00E + 00	1.05E-05	− 1.05E-03	0.00E + 00	8.58E + 03	1.54E-04	− 1.54E-02	− 1.20E-01
1.25	1.076	9.80E + 04	3.00E + 03	5.00E + 03	1.05E-05	− 1.05E-03	5.05E-04	8.58E + 03	1.54E-04	3.50E-02	− 7.00E-02
1.25	1.076	9.80E + 04	3.00E + 03	1.00E + 04	1.05E-05	− 1.05E-03	1.01E-03	8.58E + 03	1.54E-04	8.55E-02	− 1.95E-02
1.25	1.076	9.80E + 04	3.00E + 03	1.50E + 04	1.05E-05	− 1.05E-03	1.51E-03	8.58E + 03	1.54E-04	1.36E-01	3.10E-02

**Table 20:** Well data for the HOC job with the QT800 CT.

Particular	Data
Well temperature	80 °C
Oil temperature	95 °C
Material	QT800
Co efficient of thermal expansion	1.05E-5
Poisson’s ratio	0.28
Young’s modulus	3.12E + 07
Outer diameter of pipe	1.25 inch
Thickness	0.087 inch
Weight per unit length of pipe	152.86 lbs./feet

**Table 21:** CT Elongation for QT800 (HOC).

OD (d_o_) (in)	ID (d_i_) (in)	Ultimate Stress(*σ*_*u*_) (psi)	Internal Pressure (*P*_*i*)_ (psi)	Axial load (*F*_*a*_)(lbs)	Co- efficient of thermal expansion (*α*)	Thermal strain	$\text{X} = \frac{{\text{F}}_{\text{a}}}{\text{EA}}$	Y = $\frac{{\text{d}}_{\text{i}}^{2}{P}_{i}-{\mathrm{d}}_{\mathrm{o}}^{2}{P}_{o}}{{\mathrm{d}}_{\mathrm{o}}^{2}-{\mathrm{d}}_{\mathrm{i}}^{2}}$	Z=(2μ/E)*Y	ΔL/L*100	ΔL/L*100(Thermal consideration)
1.25	1.076	9.80E + 04	3.00E + 03	0.00E + 00	1.05E-05	1.58E-04	0.00E + 00	8.58E + 03	1.54E-04	− 1.54E-02	3.37E-04
1.25	1.076	9.80E + 04	3.00E + 03	5.00E + 03	1.05E-05	1.58E-04	5.05E-04	8.58E + 03	1.54E-04	3.50E-02	5.08E-02
1.25	1.076	9.80E + 04	3.00E + 03	1.00E + 04	1.05E-05	1.58E-04	1.01E-03	8.58E + 03	1.54E-04	8.55E-02	1.01E-01
1.25	1.076	9.80E + 04	3.00E + 03	1.50E + 04	1.05E-05	1.58E-04	1.51E-03	8.58E + 03	1.54E-04	1.36E-01	1.52E-01
1.25	1.076	9.80E + 04	3.00E + 03	2.00E + 04	1.05E-05	1.58E-04	2.02E-03	8.58E + 03	1.54E-04	1.86E-01	2.02E-01

### Experimental work

#### Pressure and tension limits

In this study, the Von Mises yield criterion was used to determine limit curves. The CT operations on a well with a 7-inch production casing were investigated for this. The downhole configuration included a CT with an outside diameter of 1.25 inches, an internal diameter of 1.076 inches, and a thickness of 0.087 inches. The CT’s internal pressure (*P*_*i*_) was determined using the internal pressure equation (Eq. 6 in Table 1). The difference in internal (*P*_*i*_) and external (*P*_*o*_) pressures of CT, i.e., *ΔP*, was then computed for each CT pipe, taking into account the external pressures to which they were subjected in downhole settings, i.e., (*P*_*o*_) at 0 psi, 2500 psi, and 5000 psi. Finally, ellipse-shaped limit curves were obtained by plotting *ΔP* versus *F*_*a*_ (axial force) data for the CT models QT 800, QT 900, and QT 1000. Limit curves were also generated at zero external pressure with and without taking a 6 percent (%) diametral expansion and a safety factor (S.F) into account in the current investigation. For the different CT materials studied in this study, the safety factor (S.F) for axial force, collapse region, and burst region was 0.8, 0.5, and 0.8, respectively.

#### Fatigue damage

Fatigue analysis was performed for three CT-assisted hot oil circulation (HOC) operations, as well as one for water injection and one for nitrogen shot jobs in oil wells. The wells are located in the porous media of the upper Assam basin, which produces crude oil and the reservoirs are producing**.** The first procedure was carried out on an oil well (X1) using a CT with an outer diameter of 2 inches, a thickness of 0.188 inches, and a length of 10,000 feet. Pouring hot oil into coiled tubing produces a pressure of 1000 psi. The second hot oil circulation work was carried out in a different oil well (X2) with the CT having an outside diameter of 1.25 inches, a thickness of 0.087 inches, and a tubing length of 5500 m. The coiled tubing had an internal pressure of 2000 psi, and the reel and gooseneck were both 72 inches in radius. Following that, fatigue analysis was done on a water injection job completed on an oil well ahead of acidization. The length of the water injection well (X3) was 3285 m (11,761 feet). The outside diameter was 1.25 inches, and the wall thickness was 0.087 inches. When water was pumped into the CT, it caused an internal pressure of 2500 psi. Furthermore, fatigue damage analysis was performed for another hot oil circulation project, on a well (X4) with a tubing length of 2482 m (8364 feet), an outside diameter of 1.25 inches, and a wall thickness of 0.087 inches. The circulation of heated oil produced an internal pressure of 3000 psi in this CT work. Finally, the current work on fatigue damage analysis included a nitrogen shot operation performed on an oil well (X5) with a length ranging from 1500 to 2540 m (5055–8560 feet). The outer diameter of the CTU used on the X5 is 1.25 inches, while the wall thickness is 0.087 inches. The CT produced an internal pressure of 2000 psi during the nitro shooting process. This study constructed a computer model in MATLAB software using the mathematical equations presented in Table 2 to conduct the fatigue analysis. The model was created using well data to investigate the behaviour of the CTs, i.e., QTs of 700, 800, 900, and 1000, in actual oil well conditions.

#### Temperature effect on CT elongation

The proposed CT strain-temperature correlation (Eq. 22) was used in this study to examine coiled tubing elongation for different fluid pumping temperatures. For the CTU operation, the nitrogen shooting temperature was fixed at (− ) 10 °C, while the well temperature was at 90 °C. Following that, the CT elongation study was conducted for a hot oil circulation operation. In this case, the second CT elongation study with developed correlation, the well temperature was at 80 °C, while the hot oil circulation temperature was set at 95 °C.

## Experimental results

### Pressure and tension limits

In this study, limit curves have been constructed for each of the CT listed in Table 3, with or without taking external pressure (*P*_*o*_) into account. The internal pressure, ± *P*_*i*_, was computed for all the CTs with various *F*_*a*_’s.

#### Pressure and tension limit curves for CTs at zero external pressure

**Figure 1:**
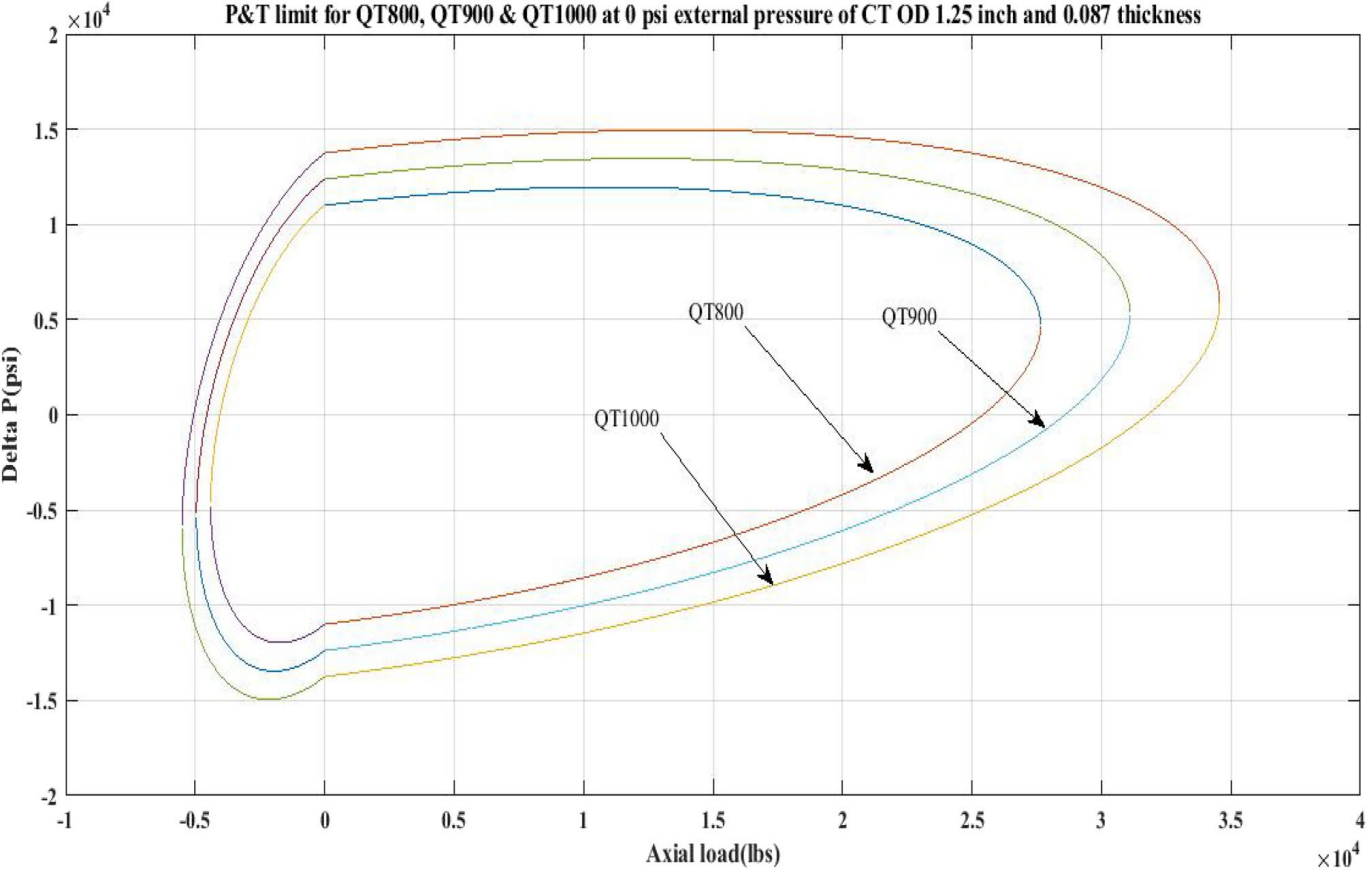
Limit curves for QT 800, QT900 and QT 1000 at zero *P*_*o*_ for the CT with 1.25 inch OD and 0.087 inch thickness.

Limit curves were generated in this work for the CTs QT800, QT900, and QT1000 by varying axial load, *F*_*a*_, and the associated *∆P* values in the MATLAB computational model. Figure 1 depicts the CT limit curves at zero external pressure. The current work estimates the internal pressure, *P*_*i*_, for the CTs by incorporating the values of various parameters in Eq. 6 of Table 1, as *β* = 6.721311, *α* = 52.89734, *γ* = 179,915.4501, *δ* = − 5.4E + 09, and, *σ*_*a*_ = 31,446.54 psi. Table 4 summarizes the internal pressure (*P*_*i*_) recorded in this investigation for an axial load (*F*_*a*_) of 10,000 lb and the related *∆P* values calculated by subtracting external pressure, *P*_*o*_, from *P*_*i*_.

The limit curve in Fig. 1 demonstrates that when subjected to a 10,000 lb axial load, QT 800 coil tubing will fail when the differential pressure (*ΔP*) exceeds 11,956.67 psi in the burst zone and 8555.45 psi in the collapse region. Furthermore, the current work implies that the *∆P* values found for QT900 are greater than the values obtained for QT800. As a result, the QT900 is less likely to fail than the QT800 when subjected to the same 100,000-pound load. In addition, as shown in Fig. 1, the area under the collapse region for QT800 is 1.5 square units. In the current study, this area was estimated by splitting the collapse region into 0.5 × 0.5 grids. Similarly, the area covered by the burst region is approximately 2.75 square units. The collapse region for QT900 is 2.25 square units in size, and the burst region is 3.5 square units in size. For QT1000, the area under the collapse region is 3.25 square units, whereas the area under the burst region is 5 square units. Because the area under the collapse zone for each CT material is less than the area under the burst region, coil tubing is more prone to collapse failure than burst failure.

#### Pressure and tension limit curves for different external pressure

**Figure 2:**
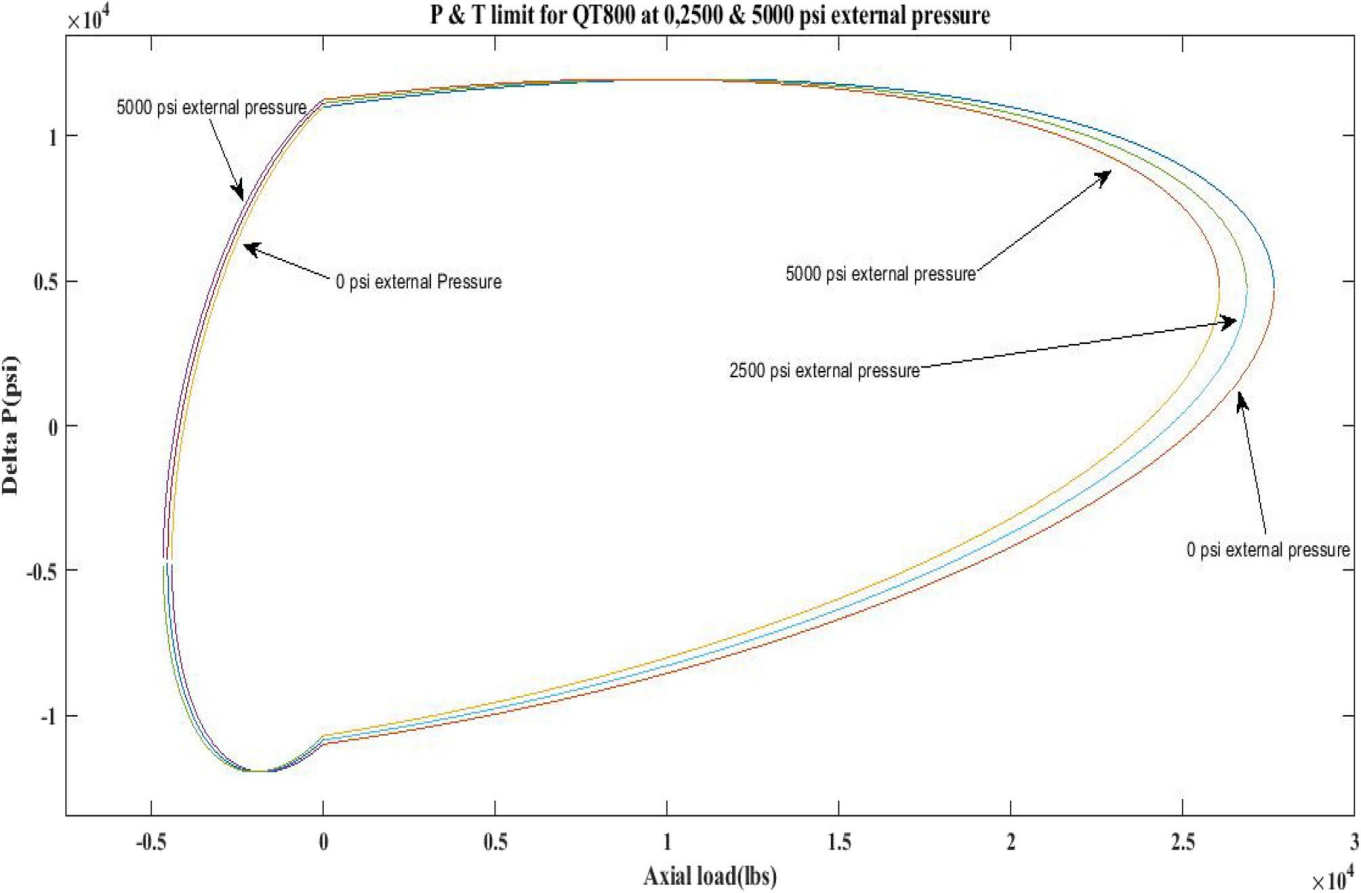
Limit curves for QT 800 at 0, 2500 psi, and 5000 psi external pressures.

**Figure 3:**
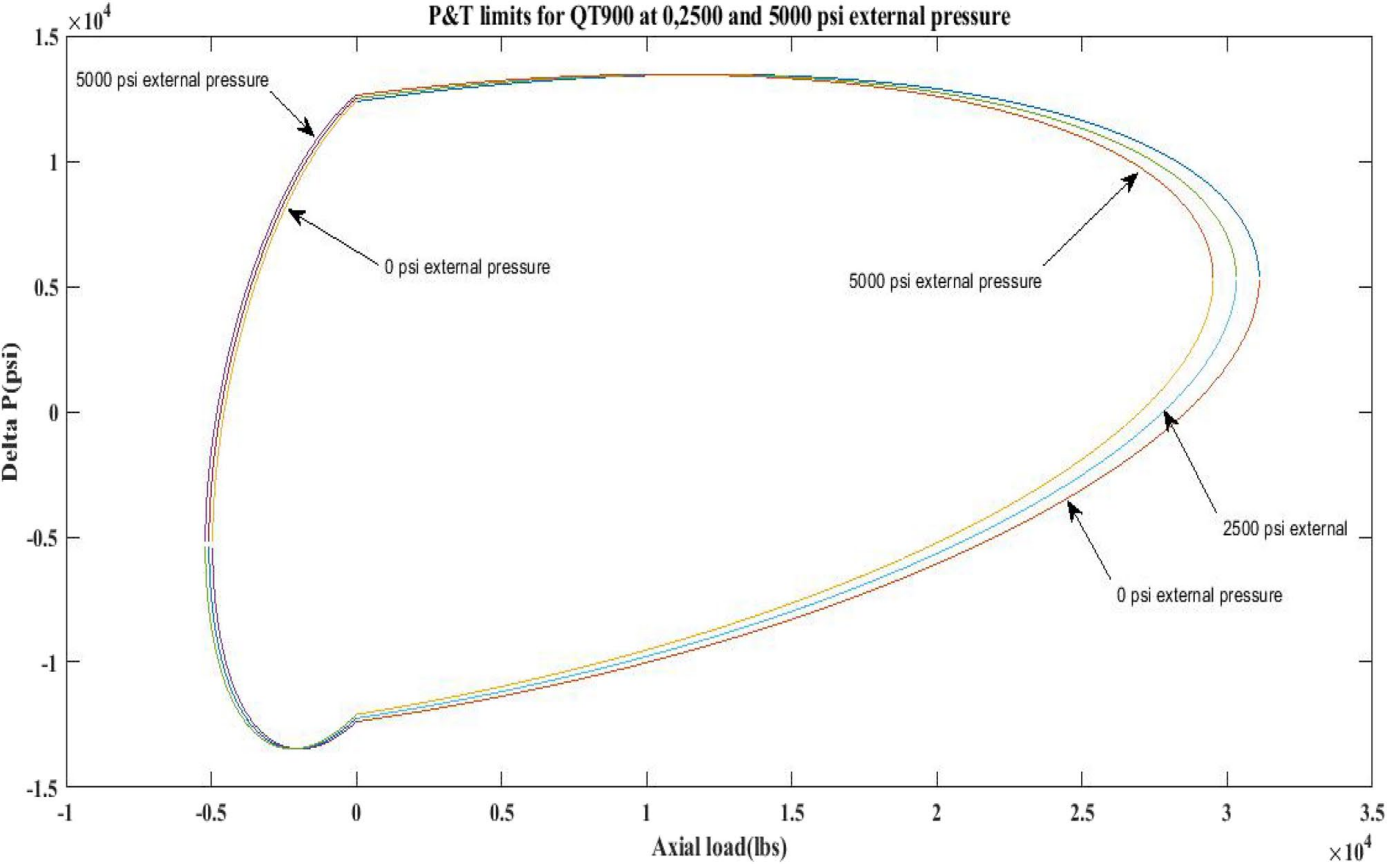
Pressure & Tension limits for QT 900 at 0, 2500 psi and 5000 psi external pressures.

**Figure 4:**
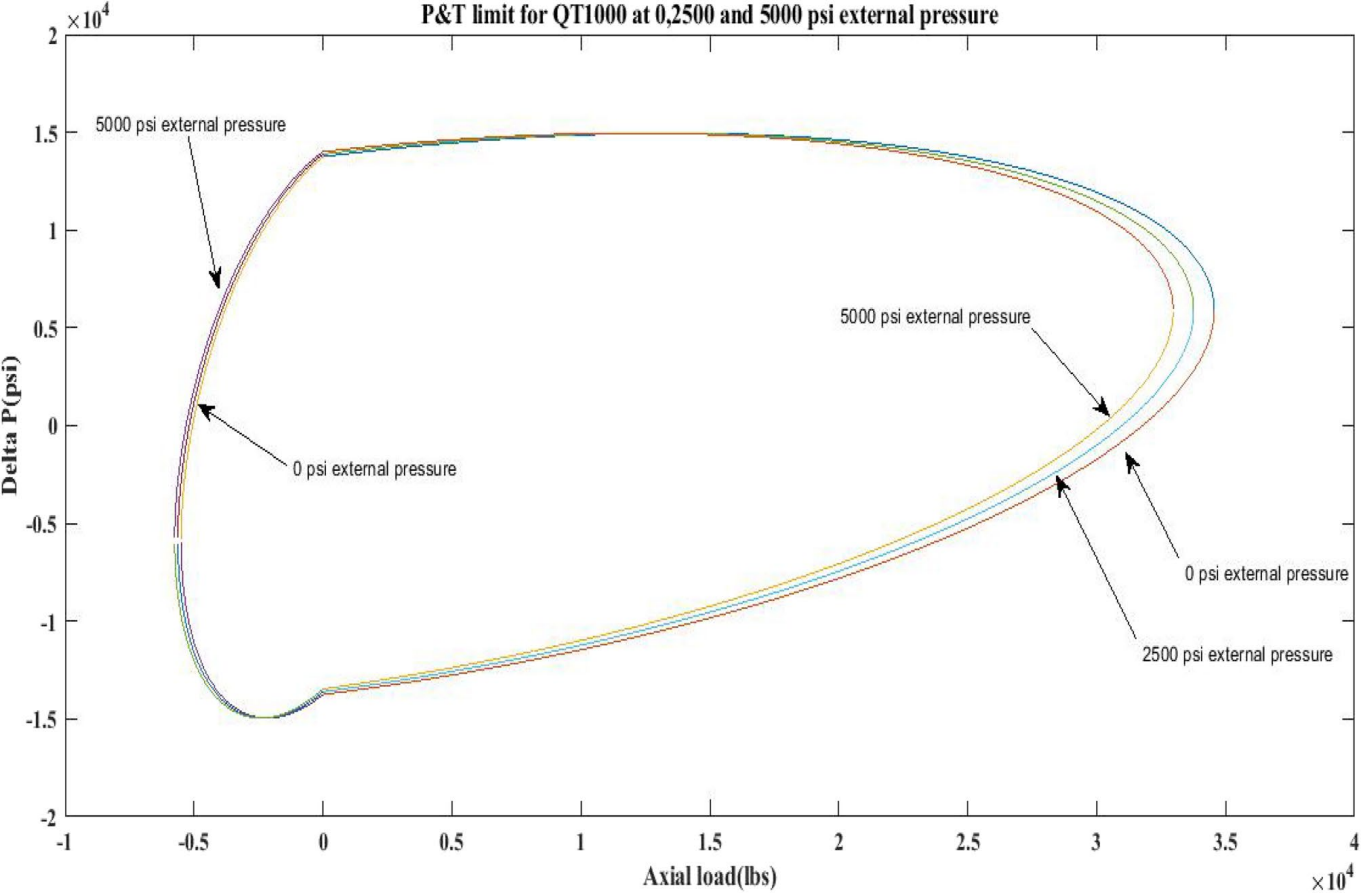
Pressure and tension limits for QT 1000 at 0, 2500 psi and 5000 psi external pressure.

**Figure 5:**
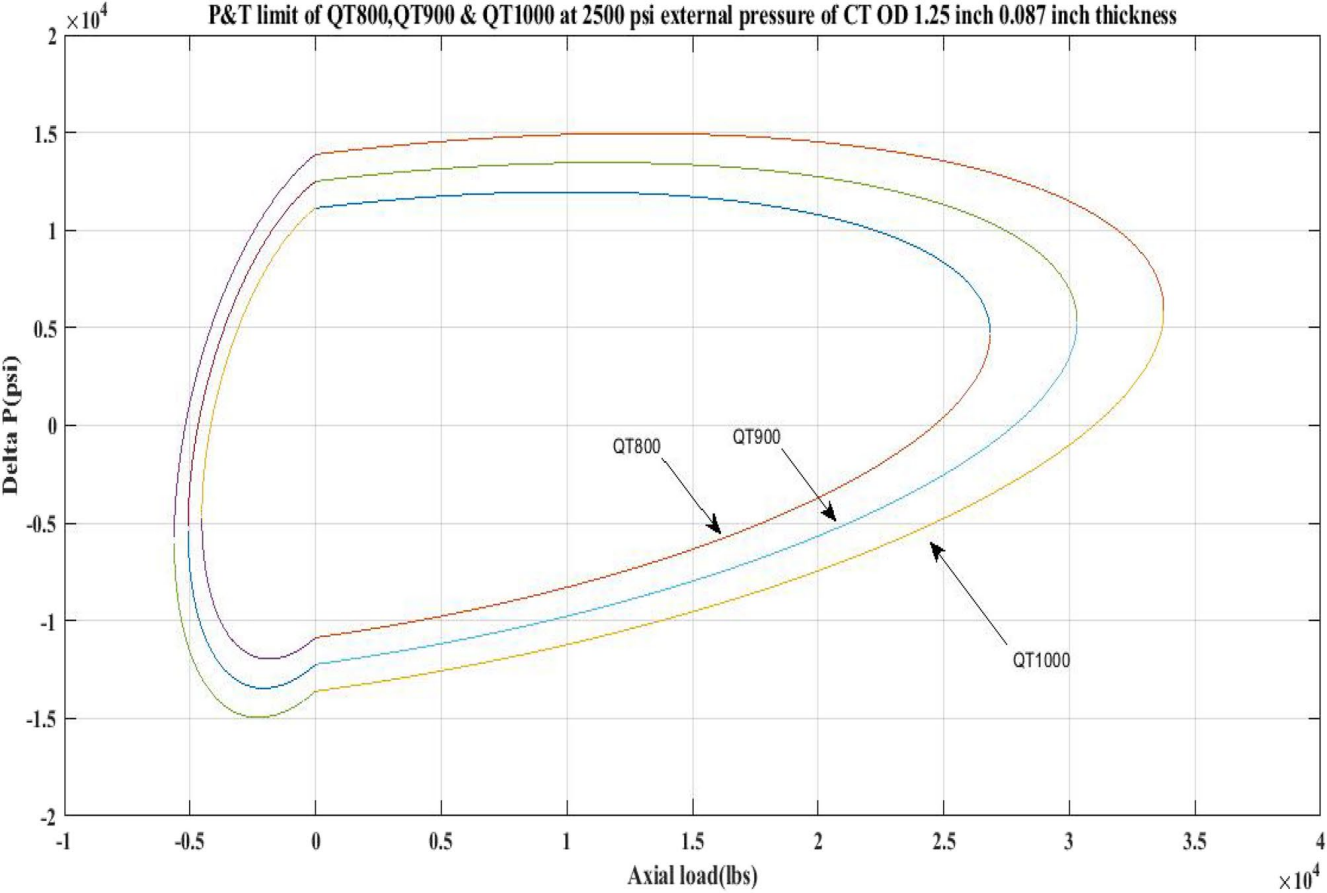
Pressure and tension limits for QT 800, QT900 and QT 1000 at 2500 psi external pressure.

**Figure 6:**
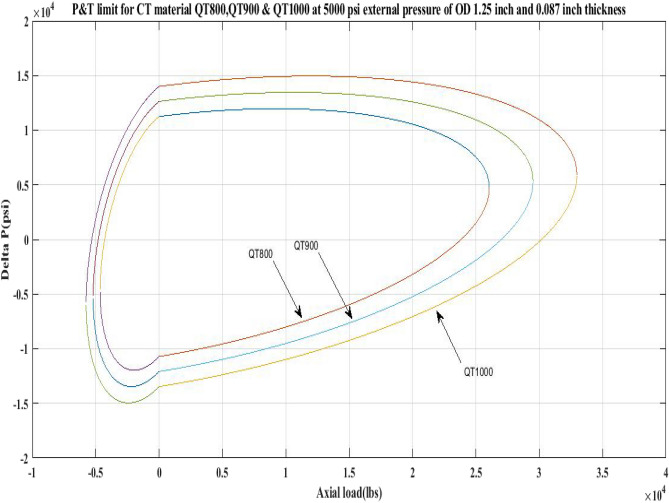
Pressure and tension limits for QT 800, QT900 and QT 1000 at 5000 psi external pressure.

This work generated P and T limit curves for CTs QT800, QT900, and QT1000 for three different external pressure settings, namely *P*_*o*_ = 0 psi, 2500 psi, and 5000 psi. The yield limit for CTs was then determined using Eq. 6 (Table 1) and sub-Eqs. 6a, 6b, 6c, 6d, and 1 to determine *β*, *α*, *γ* as well as *δ* and *σ*_*a*_. Following that, the limiting values of CTs under an axial load, *F*_*a*_ of 25,000 lb and 10,000 lb, have been analysed. Figures 2, 3, 4, 5, 6, and Table 5 depict the limit curve behaviour of CTs, with *ΔP* values at various *P*_*i*_ and *P*_*o*_.

#### Pressure and tension limit curves considering 6% diametric growth

**Figure 7:**
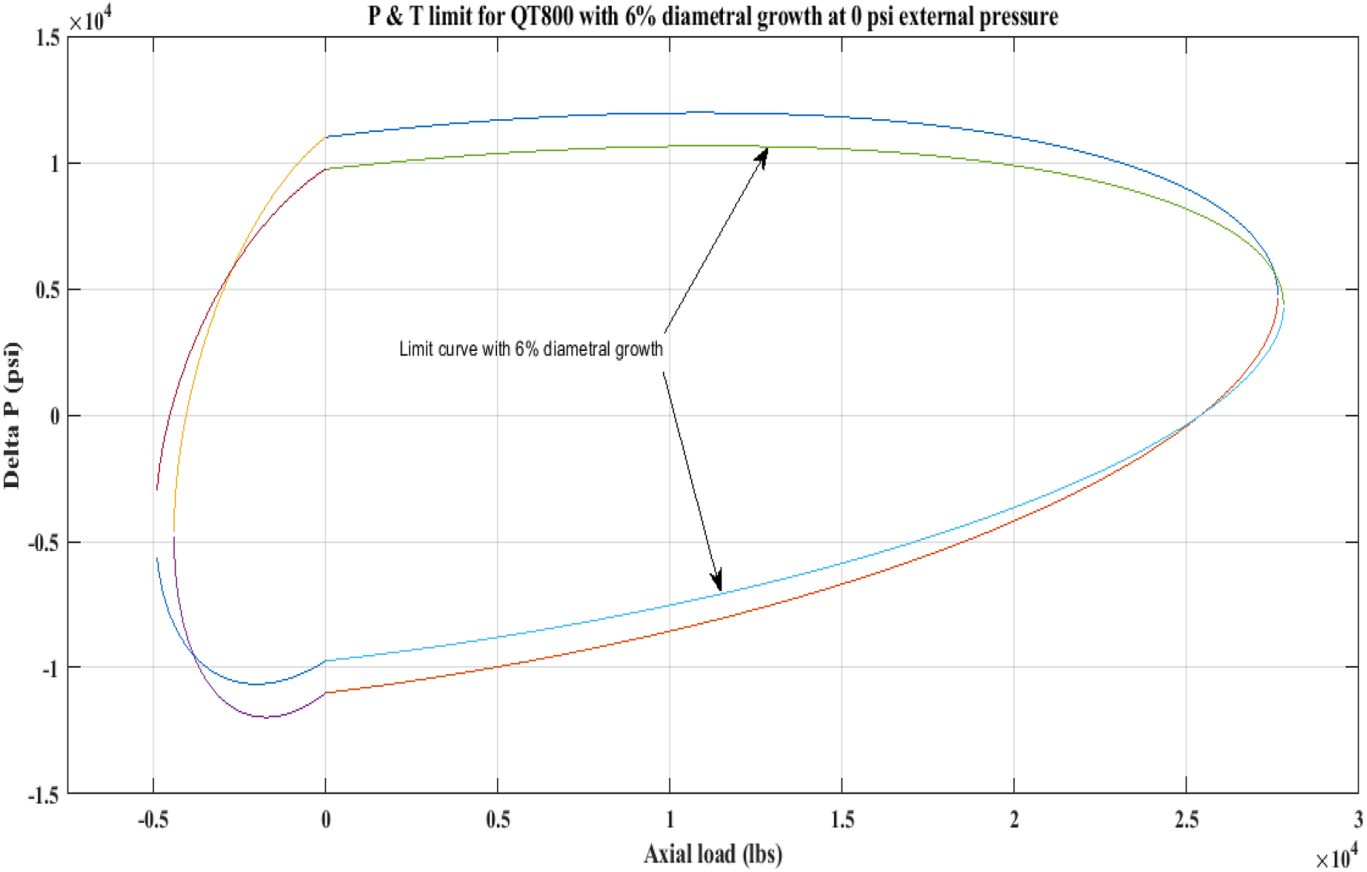
P and T limit with diameter growth for QT800 at 0 *P*_*o*_.

**Figure 8:**
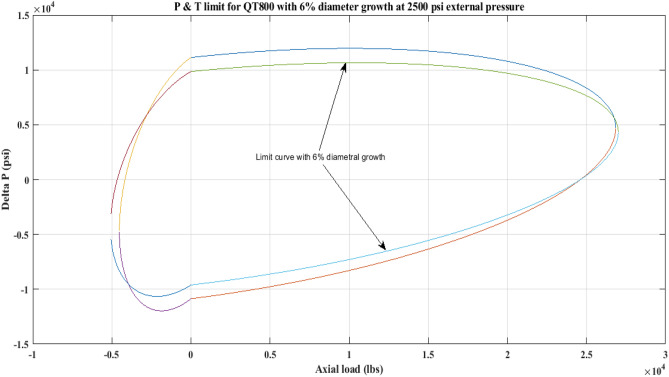
P and T limit with diameter growth for QT800 at 2500 psi *P*_*o*_.

**Figure 9:**
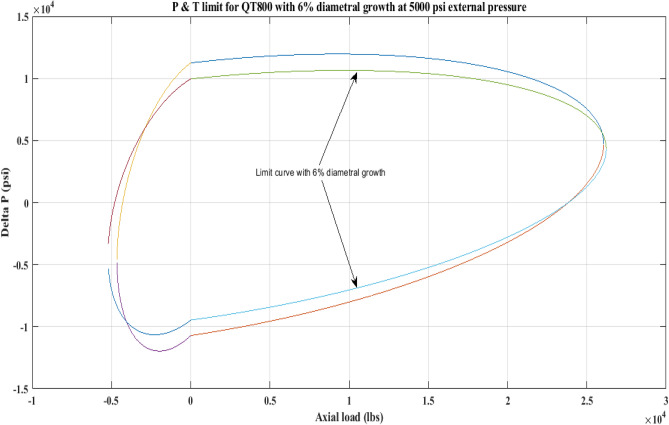
P and T limit with diameter growth for QT800 at 5000 psi *P*_*o*_.

**Figure 10:**
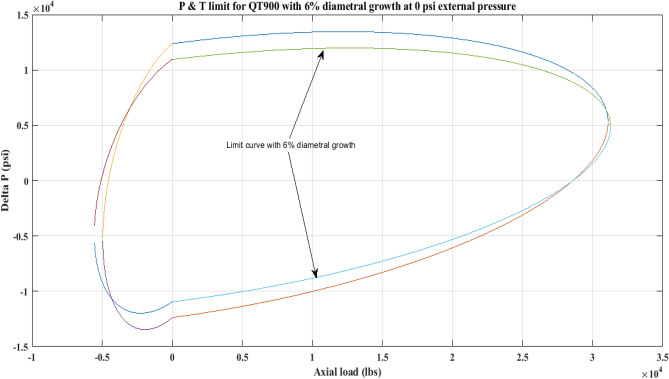
P and T limit with diameter growth for QT 900 at 0 *P*_*o*_.

**Figure 11:**
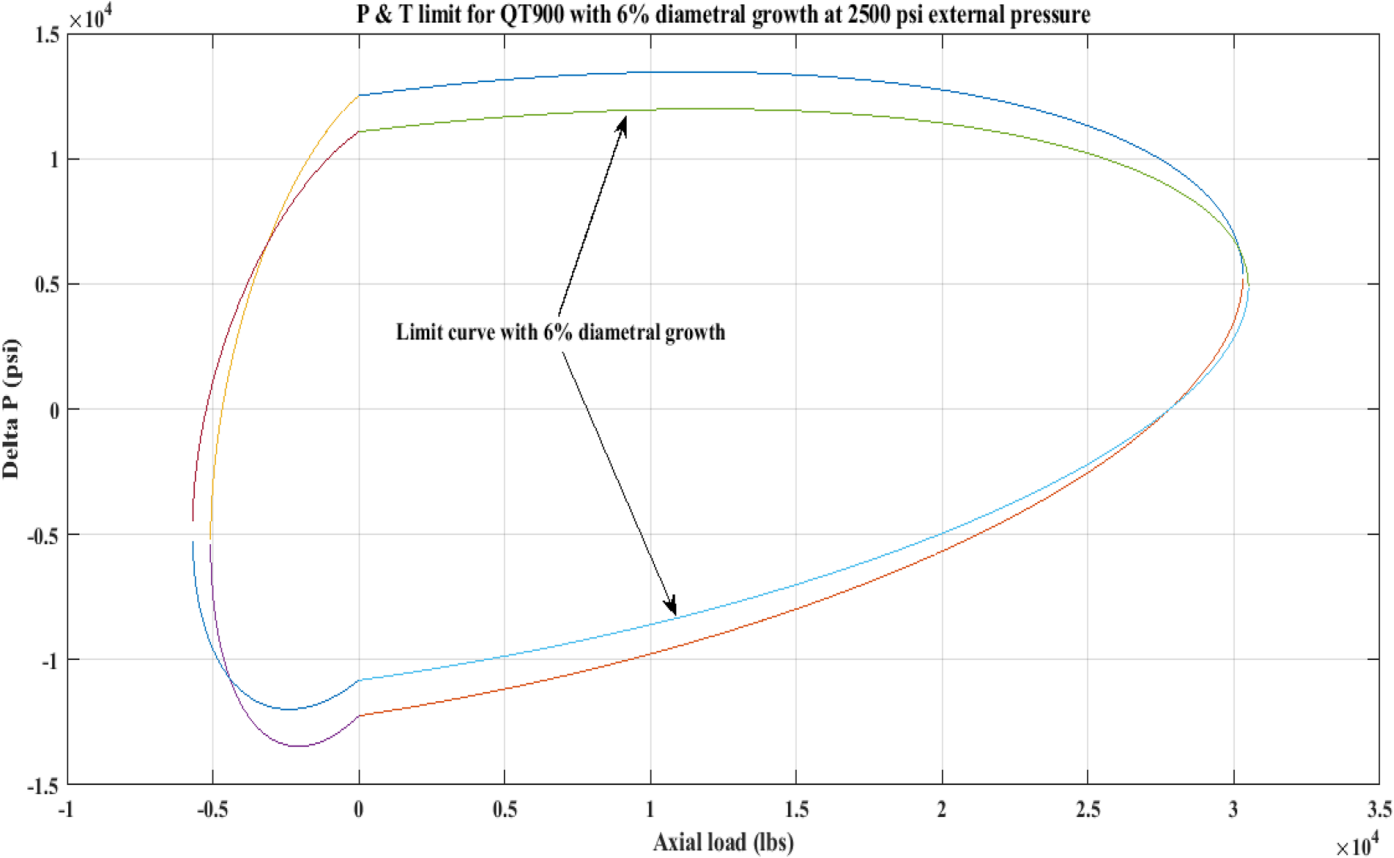
P and T limit with diameter growth for QT 900 at 2500 psi *P*_*o*_.

**Figure 12:**
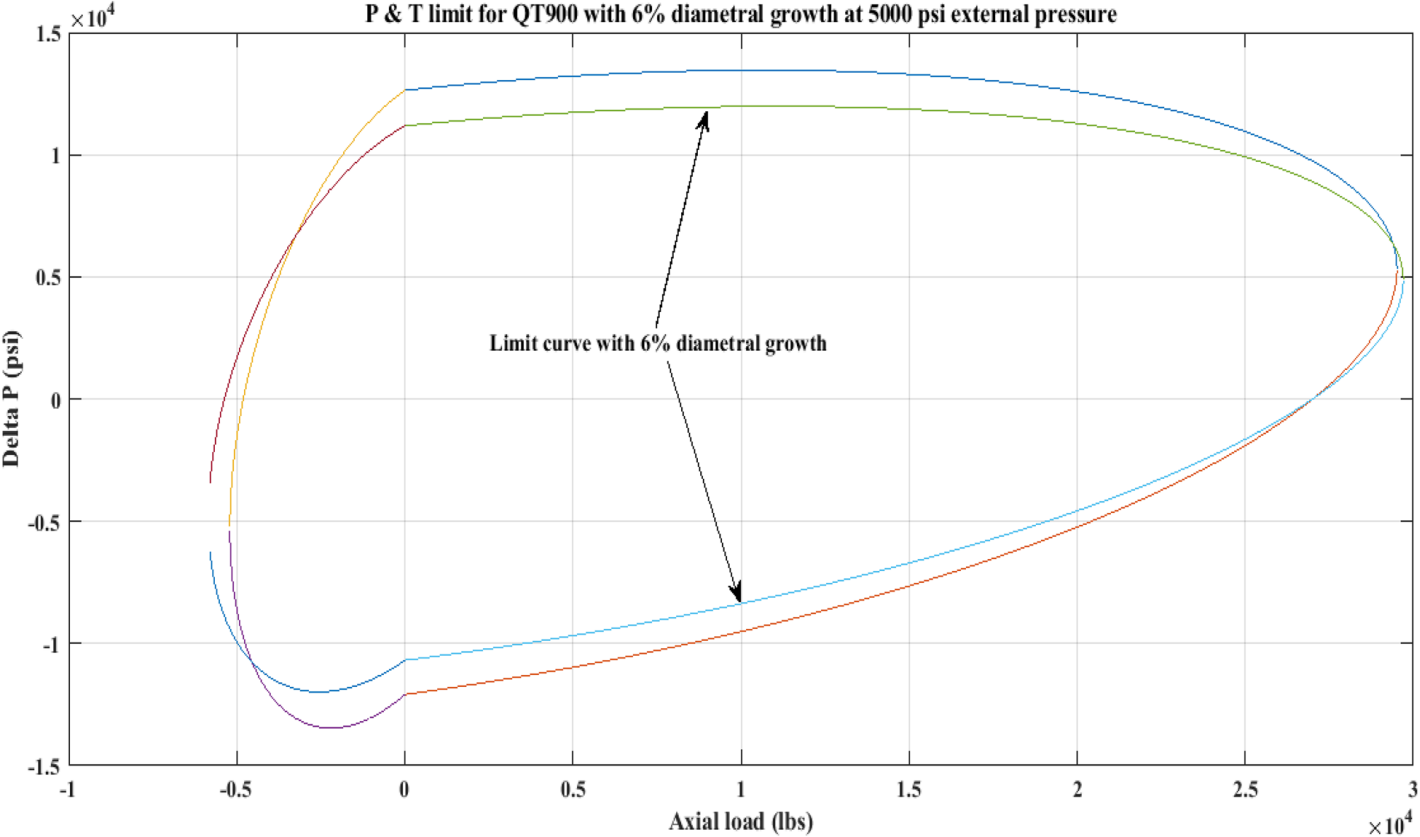
P and T limit with diameter growth for QT 900 at 5000 psi *P*_*o*_.

**Figure 13:**
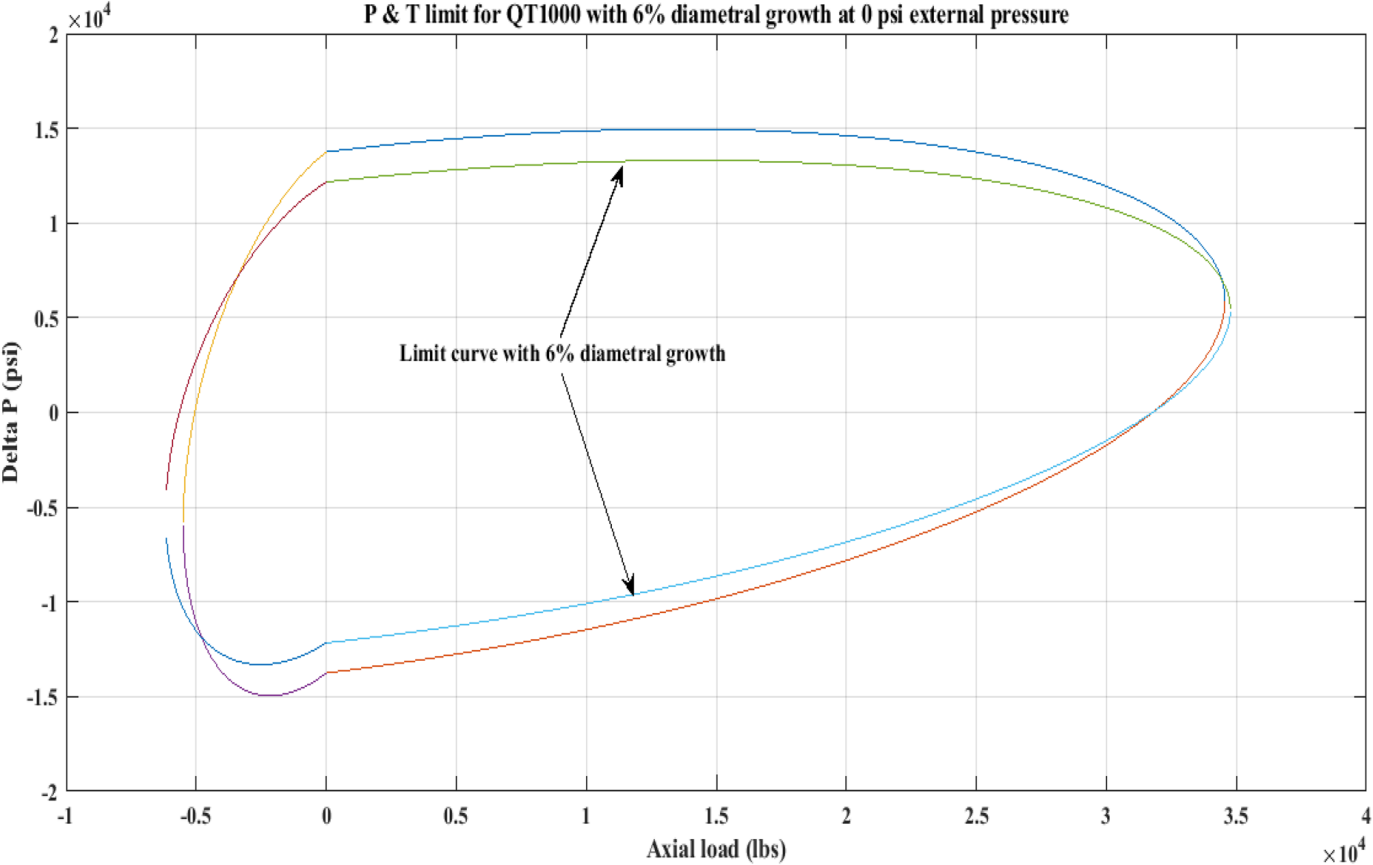
P and T limit with diameter growth for QT 1000 at 0 *P*_*o*_.

**Figure 14:**
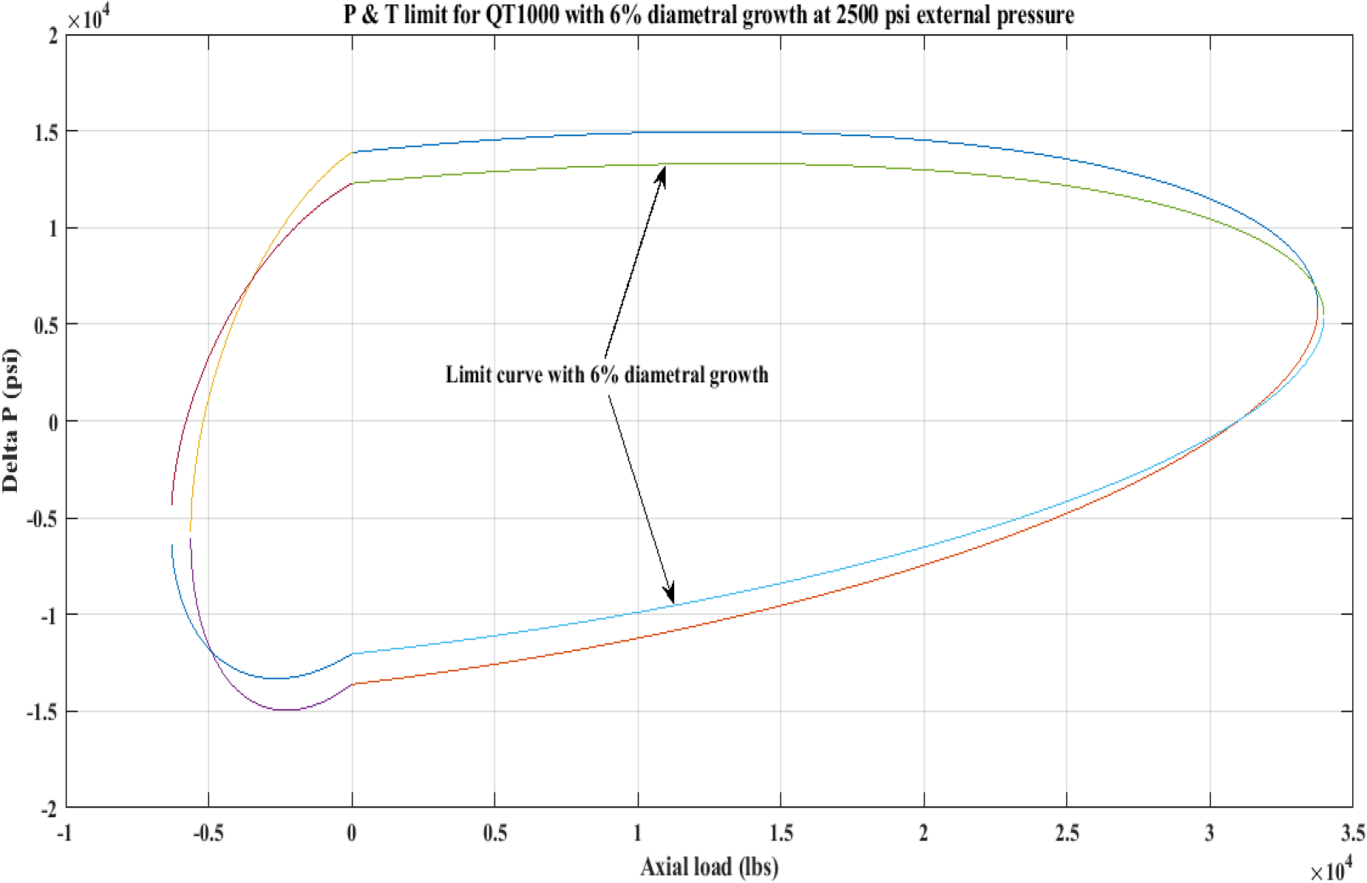
P and T limit with diameter growth for QT 1000 at 2500 psi *P*_*o*_.

**Figure 15:**
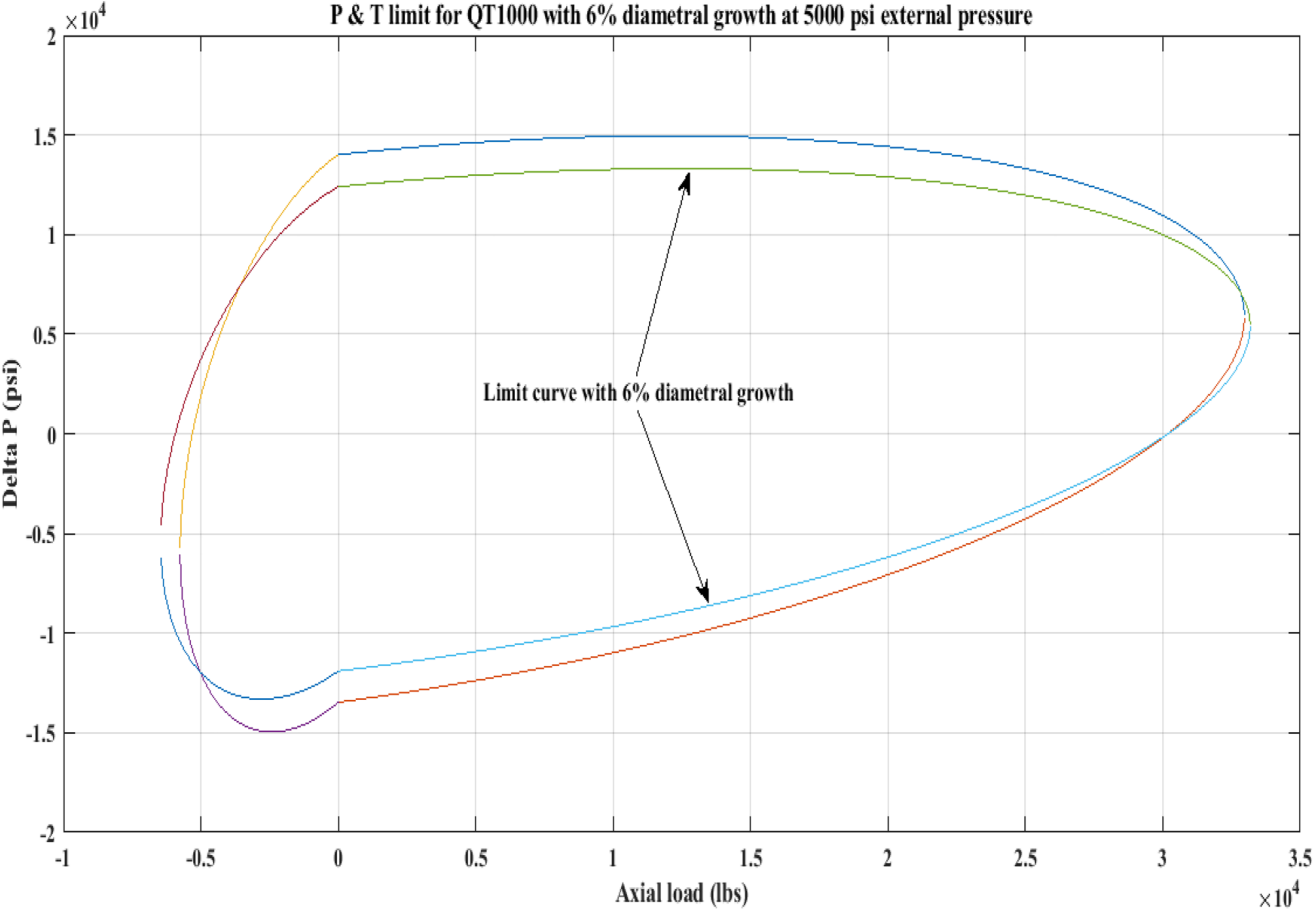
P and T limit with diameter growth for QT 1000 at 5000 psi *P*_*o*_.

In this work, the effect of increasing diameter on the P and T limit behaviour of coiled tubing was explored. When a CT’s diameter expands, its operating limit is reduced. A piece of CT should be discarded if its diameter exceeds the permissible expansion limit. The P and T limit curves of CTs QT800, QT900, and QT1000 were established in this study, taking into account a 6% diameter expansion for the CT materials. Following that, a comparison of the P and T limit behaviour of the CTs was made without taking diametric growth into account. The diameter expansion and subsequent impact on the working limit were analysed for varying external pressures, namely, *P*_*o*_ = 0 psi, 2500 psi, and 5000 psi, and an axial load, *F*_*a*_, of 10,000 lb for each of the CTs. Figures 7, 8, 9, 10, 11, 12, 13, 14, 15 illustrate the limit curves with and without a 6% diameter expansion on the nominal diameter of the coiled tubing. Table 6 shows the P and T behaviour of the three CT materials, QT800, QT900, and QT1000, with and without considering the effect of CT expansion or increase of 6% of nominal diameter.

All of the limit curves in Figs. 7, 8, 9, 10, 11, 12, 13, 14, 15 show that the *∆P* values for CT with a diameter increase are less than the *∆P* values for CT without diameter growth. When a diameter increase is considered, the working limit of a CT pipe decreases.

Table 5 shows that when the external pressure rises, the operating limit of CTs for their *ΔP* burst falls under an axial load of 25,000 lb. Furthermore, for all CTs, the area under the collapse region is smaller than the area under the burst region. Because the area under the collapse region was determined to be less than the area under the burst region for each of the CT materials QT800, QT900, and QT1000, the limit curves shown in Figs. 2, 3, 4, 5, 6, and imply that coil tubing is more prone to collapse failure than burst failure when subjected to external forces. Furthermore, as the axial load on CTs increases, the working limit of CTs for burst pressure decreases with increasing external pressure.

#### Pressure and tension limit curves considering safety factor (SF)

**Figure 16:**
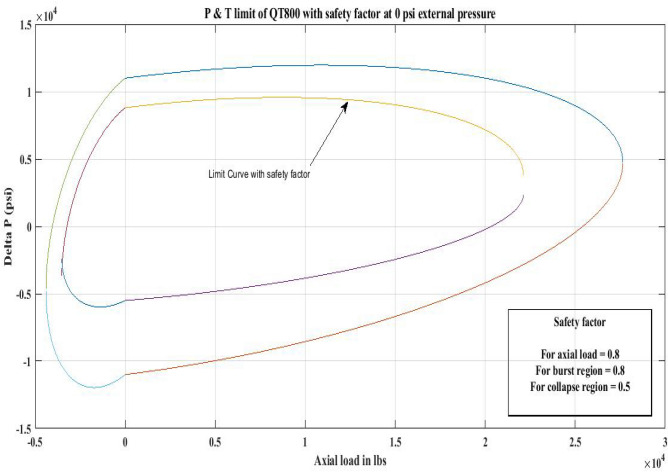
P and T limit with safety factor for QT 800 at 0 *P*_*o*_.

**Figure 17:**
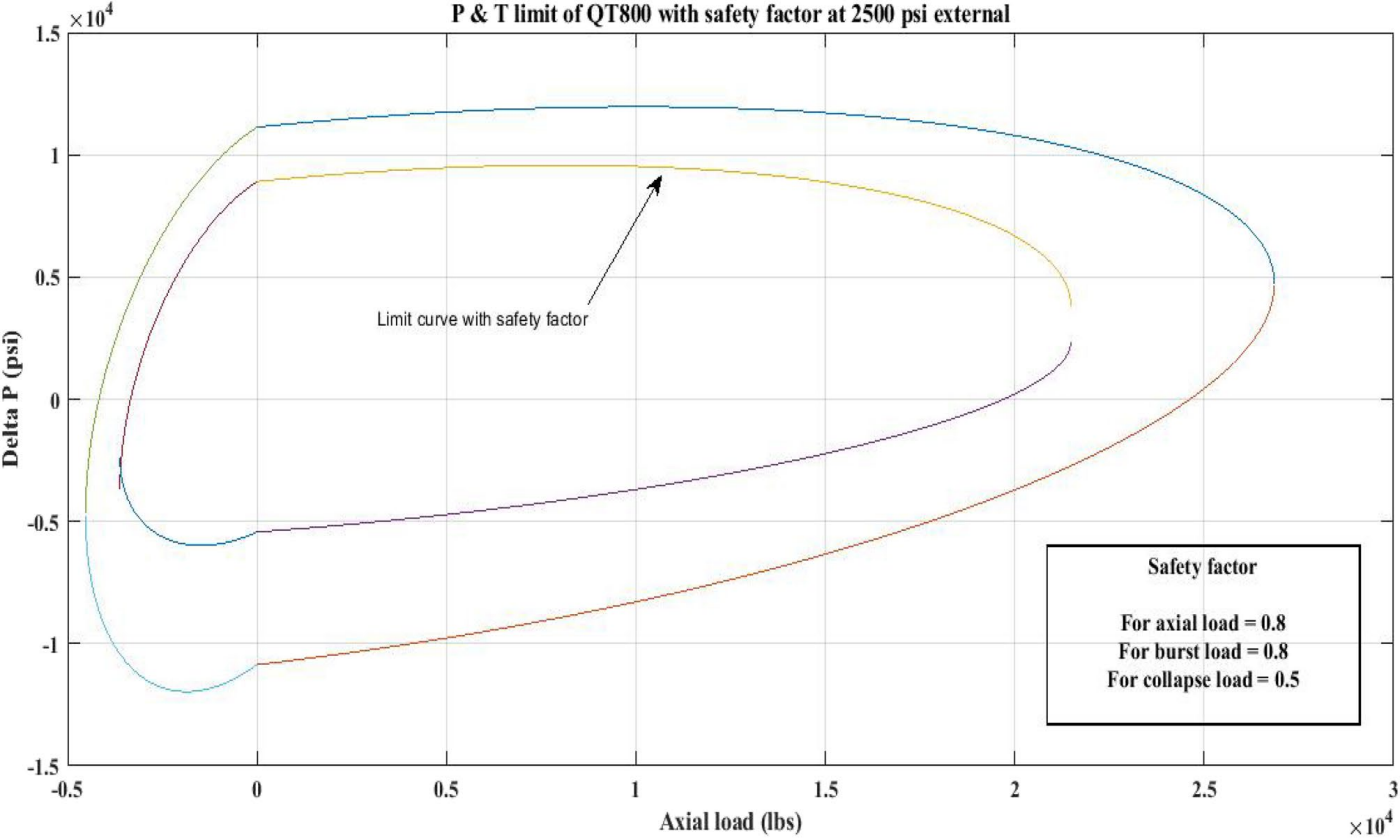
P and T limit with safety factor for QT 800 at 2500 psi *P*_*o*_.

**Figure 18:**
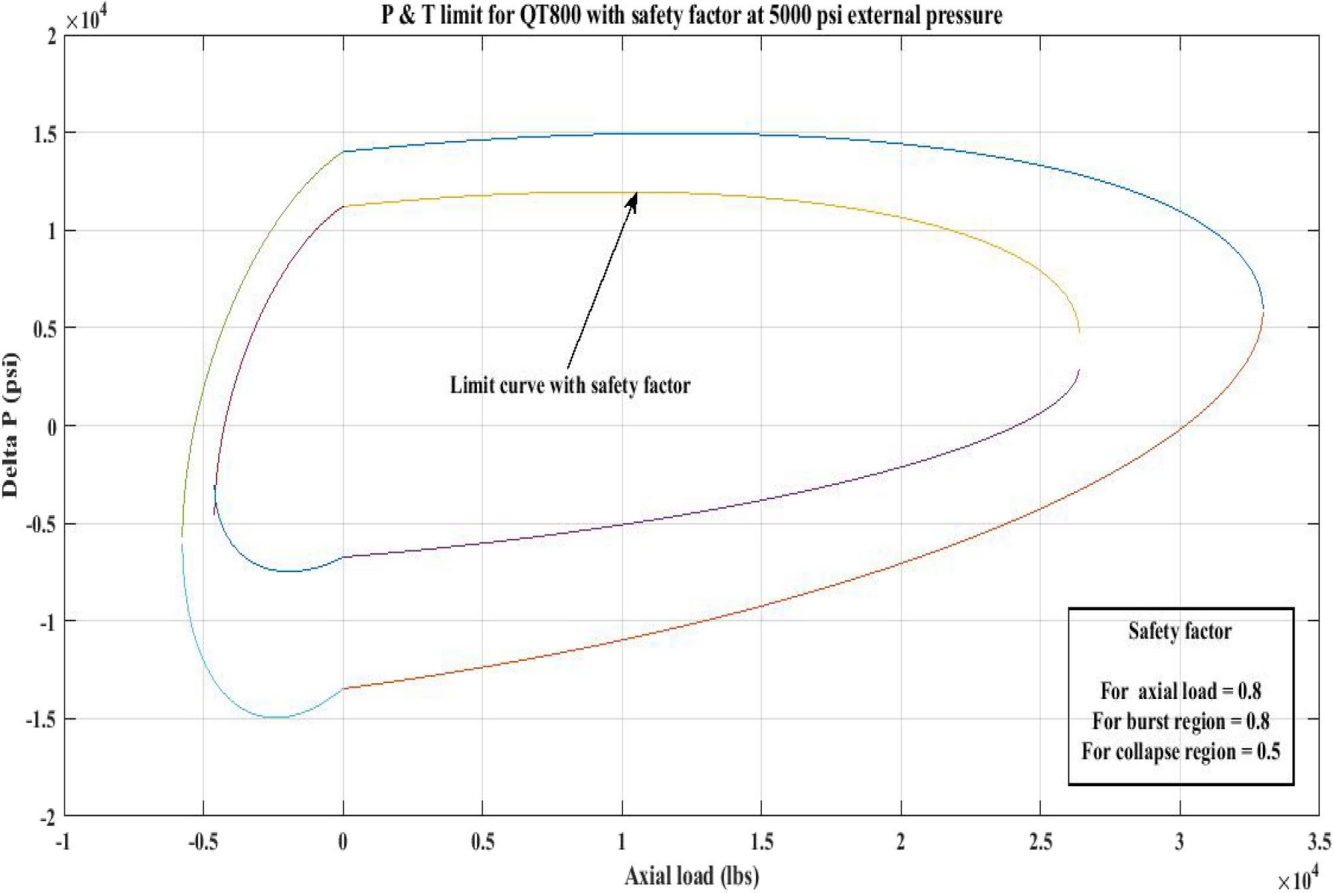
P and T limit with safety factor for QT800 at 5000 psi *P*_*o*_.

**Figure 19:**
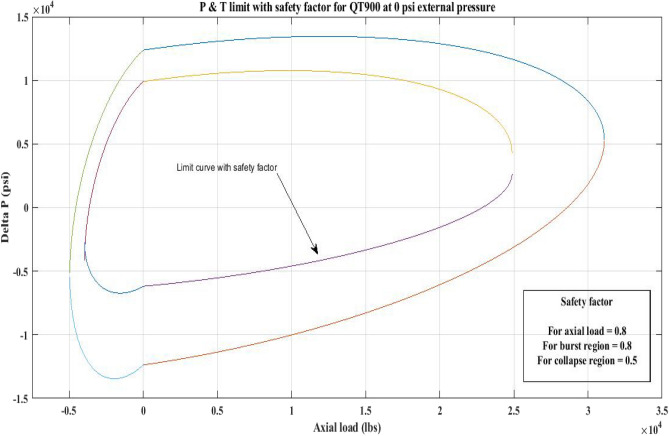
P and T limit with safety factor for QT 900 at 0 *P*_*o*_.

**Figure 20:**
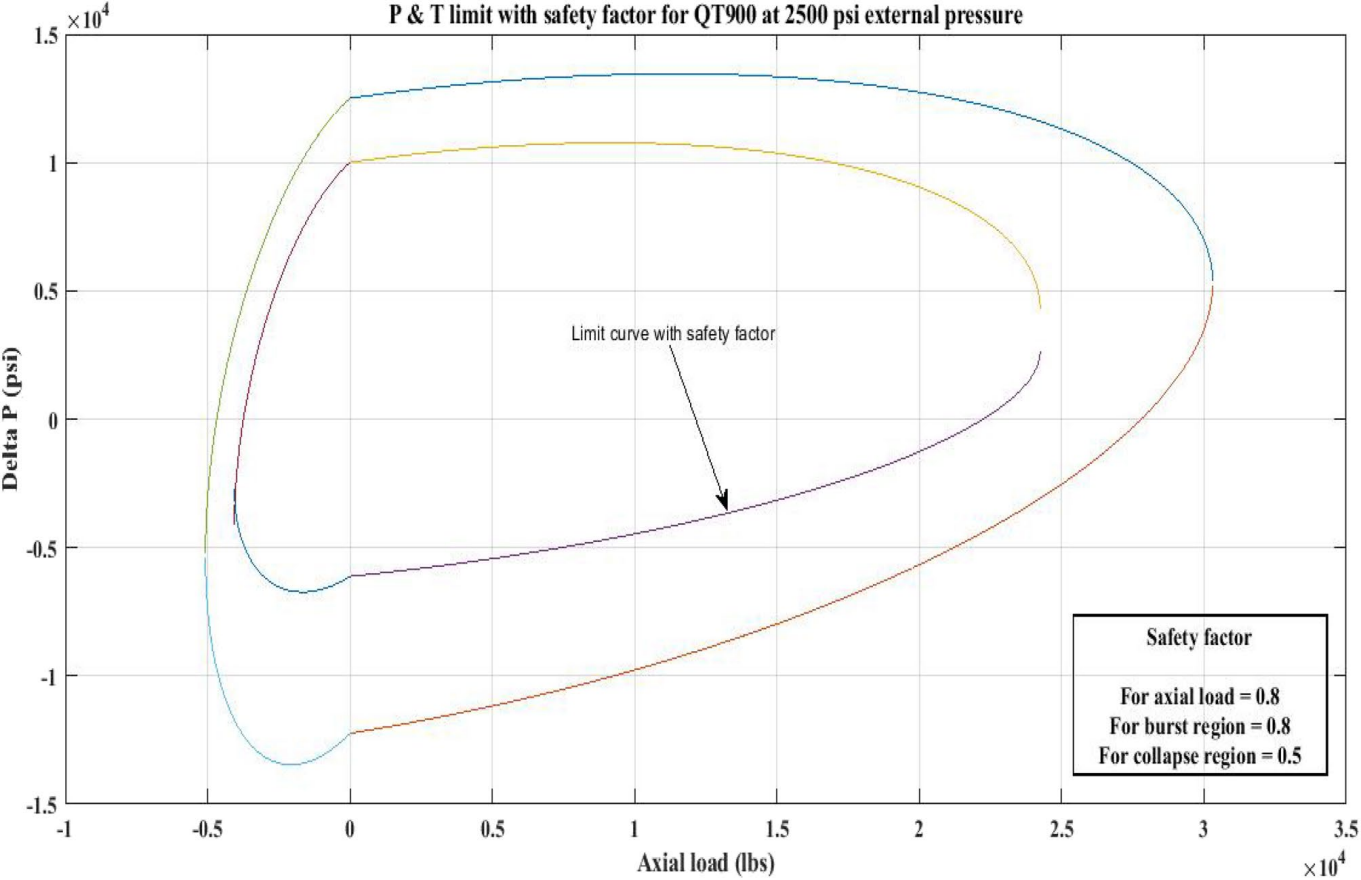
P and T limit with safety factor for QT 900 at 2500 psi *P*_*o*_.

**Figure 21:**
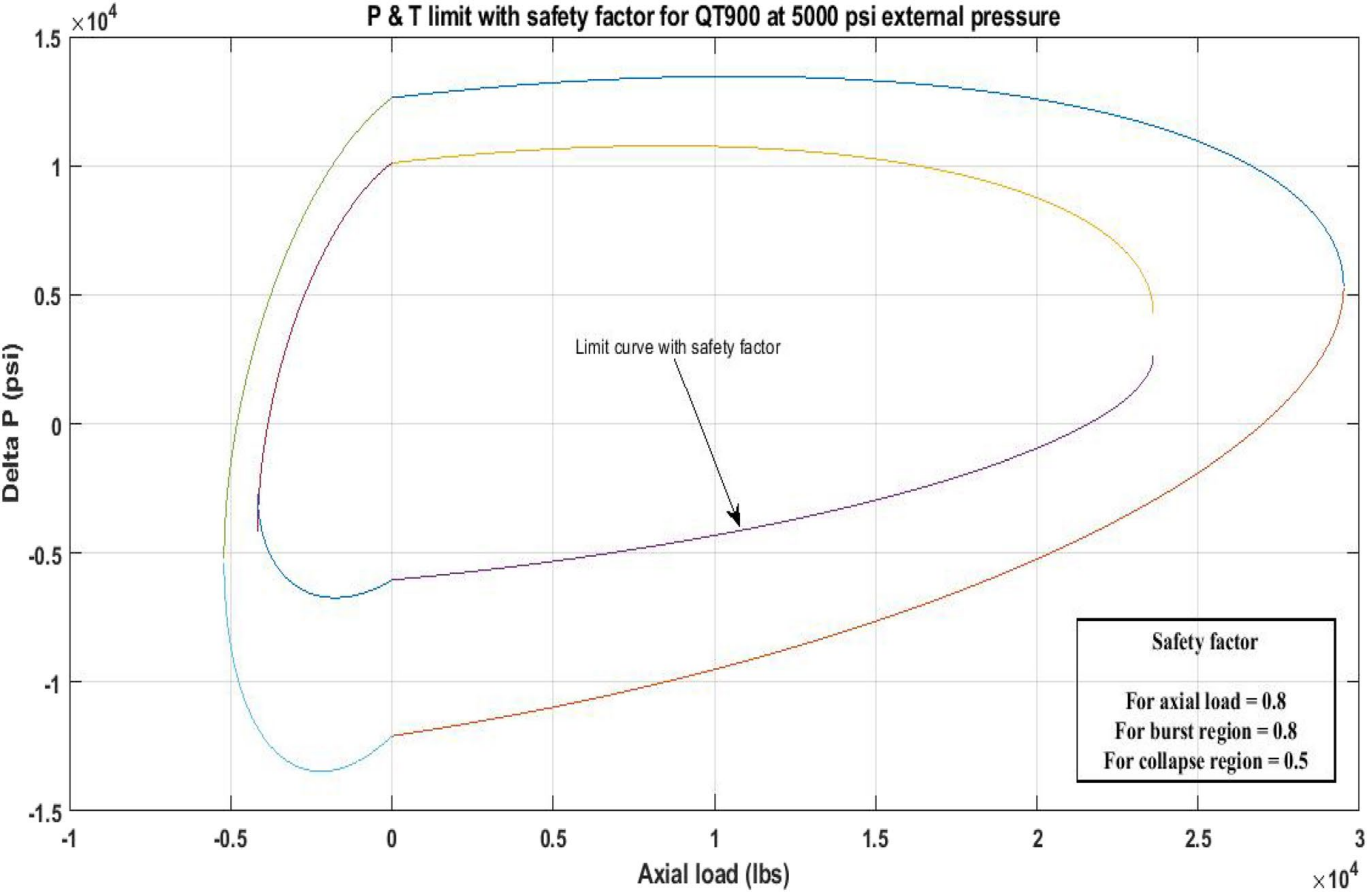
P and T limit with safety factor for QT 900 at 5000 psi *P*_*o*_.

**Figure 22:**
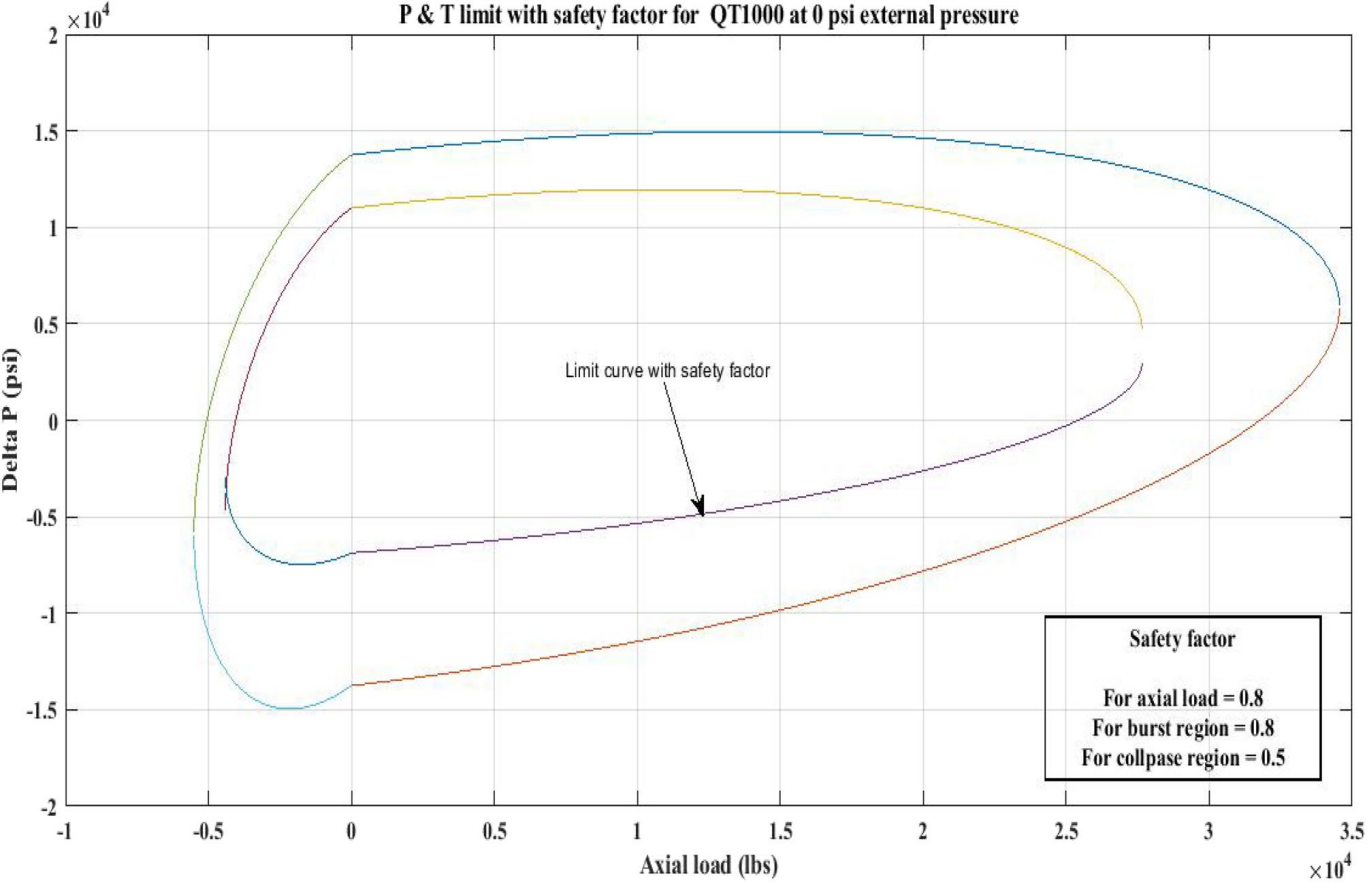
P and T limit with safety factor for QT 1000 at 0 *P*_*o*_.

**Figure 23:**
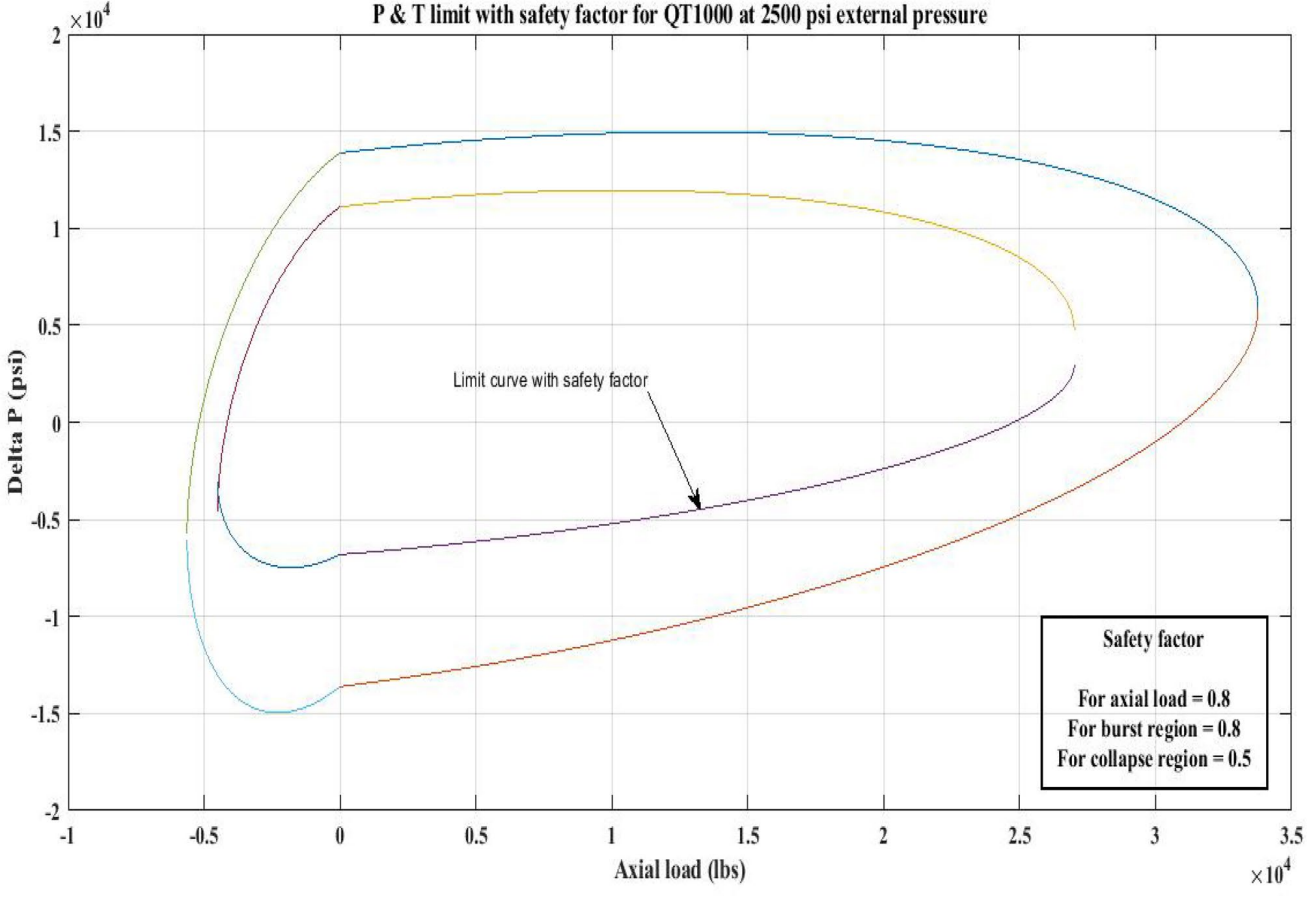
P and T limit with safety factor for QT 1000 at 2500 psi *P*_*o*_.

**Figure 24:**
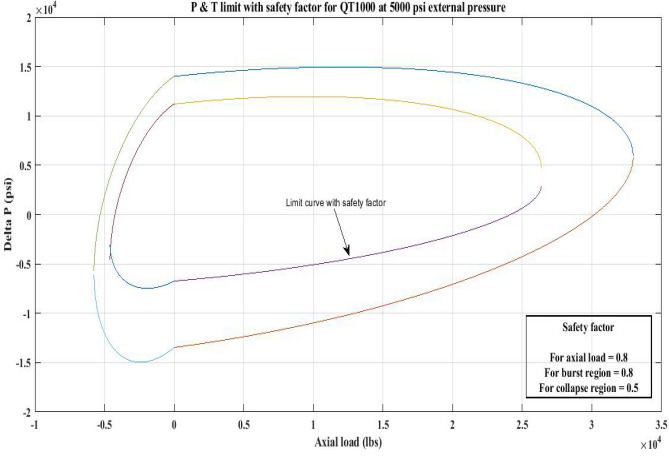
P and T limit with safety factor for QT 1000 at 5000 psi *P*_*o*_.

New limit curves for CTs, QT800, QT900, and QT1000 were created in this study considering an SF for both axial load, *F*_*a*_ and *ΔP* for burst and collapse pressure. In the current investigation, the safety factor was calculated by multiplying *F*_*a*_ and *ΔP* (burst) by 80 percent and *ΔP* (collapse) by 50 percent^6^. In order to account for the ovality impact, a greater safety factor was applied to the collapse region in the current work. Figures 16, 17, 18, 19, 20, 21, 22, 23, 24 depict the inner-working limit curve with a safety factor. Table 7 provides the values of *ΔP* with and without taking into account the safety factor calculated with the *F*_*a*_ set at 10,000 lb for each CT material.

### Fatigue damage

#### Fatigue damage of QT 700, QT 800, QT900 and QT1000 observed for 1000 psi circulation pressure (Hot oil)

Fatigue damage was estimated for the CT materials in this study based on the field data presented in Table 8 for HOC operations performed in the porous media of the upper Assam basin.

**Figure 25:**
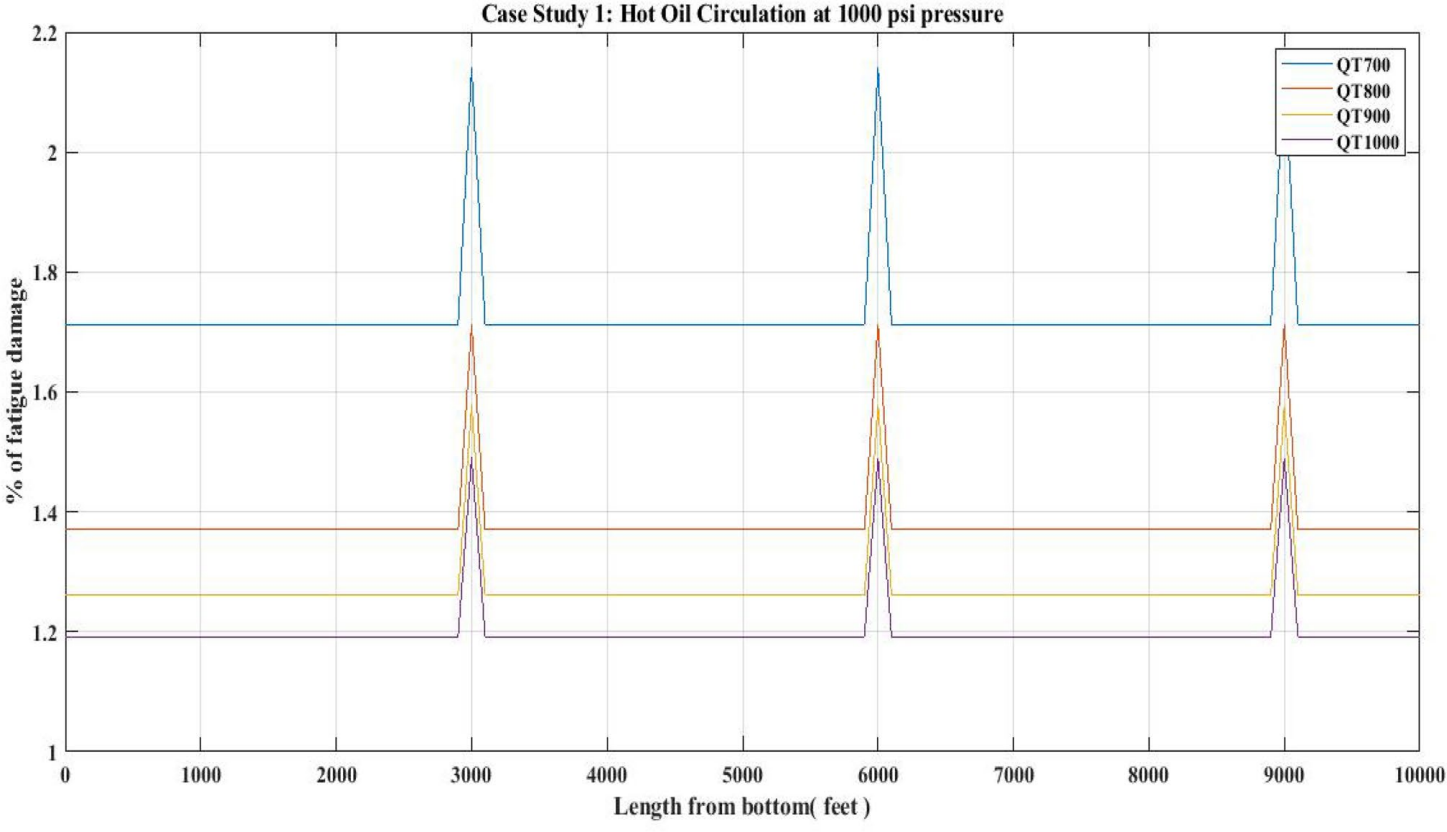
Percentage of fatigue damage with respect to hot oil circulation at 1000 psi pressure.

The current approach, as shown in Fig. 25, uses the Avakov model to calculate fatigue damage. According to Fig. 25, maximum fatigue damage has occurred at the bias welding regions, which is approximately 2.18% for QT700 CT. Table 9 compares the fatigue damage encountered during the HOC task performed with the CTs.

#### Fatigue damage of QT 700, QT 800, QT900 and QT1000 observed for 2000 psi circulation pressure (Hot oil)

In this study, the CT fatigue damage in relation to HOC activity was calculated. Table 10 displays the well data for the hot oil circulation job conducted at a circulation pressure of 2000 psi.

**Figure 26:**
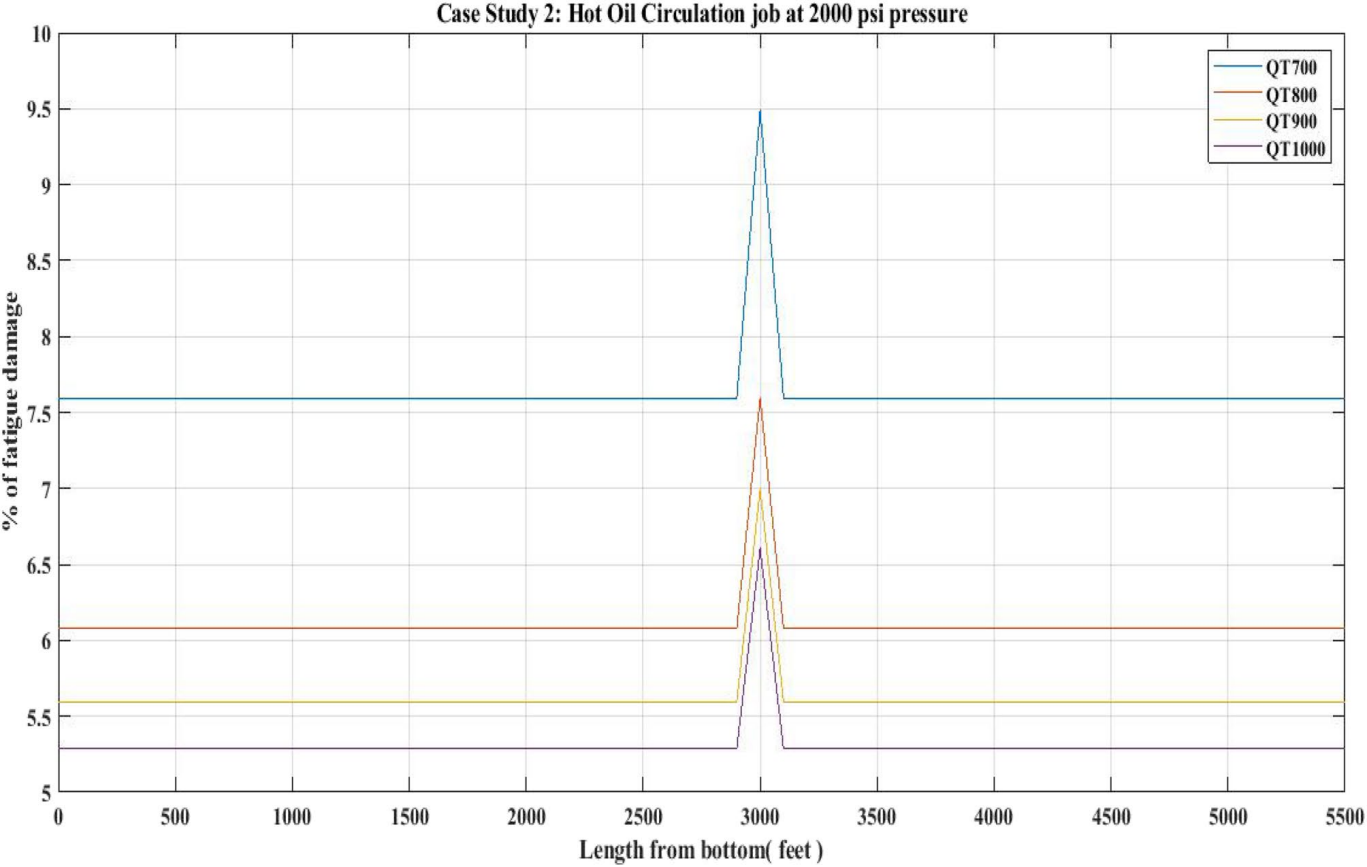
Percentage of fatigue damage with respect to hot oil circulation at 2000 psi pressure.

The fatigue damage of CT is depicted in Fig. 26 based on the results obtained with the computational analysis performed using MATLAB. According to the results in Fig. 26, fatigue damage rose to a maximum of 9.5% for the CT QT700 at its welded part. This increase in fatigue damage for the QT700 was caused by the circulation pressure being two times greater than the first circulation pressure. Furthermore, the OD of the CT pipe used for the HOC job was 1.25 inches, which was higher than the OD of the CT pipe used for the first HOC job. Table 11 compares the fatigue damage values determined for the various CTs.

#### Fatigue damage of QT 700, QT 800, QT900 and QT1000 observed for 2500 psi circulation pressure (water injection)

The fatigue damage to the CTs was explored in this investigation for the water injection job done in the porous media of the upper Assam Basin. Table 12 shows the CT data that was used to estimate the fatigue damage.

**Figure 27:**
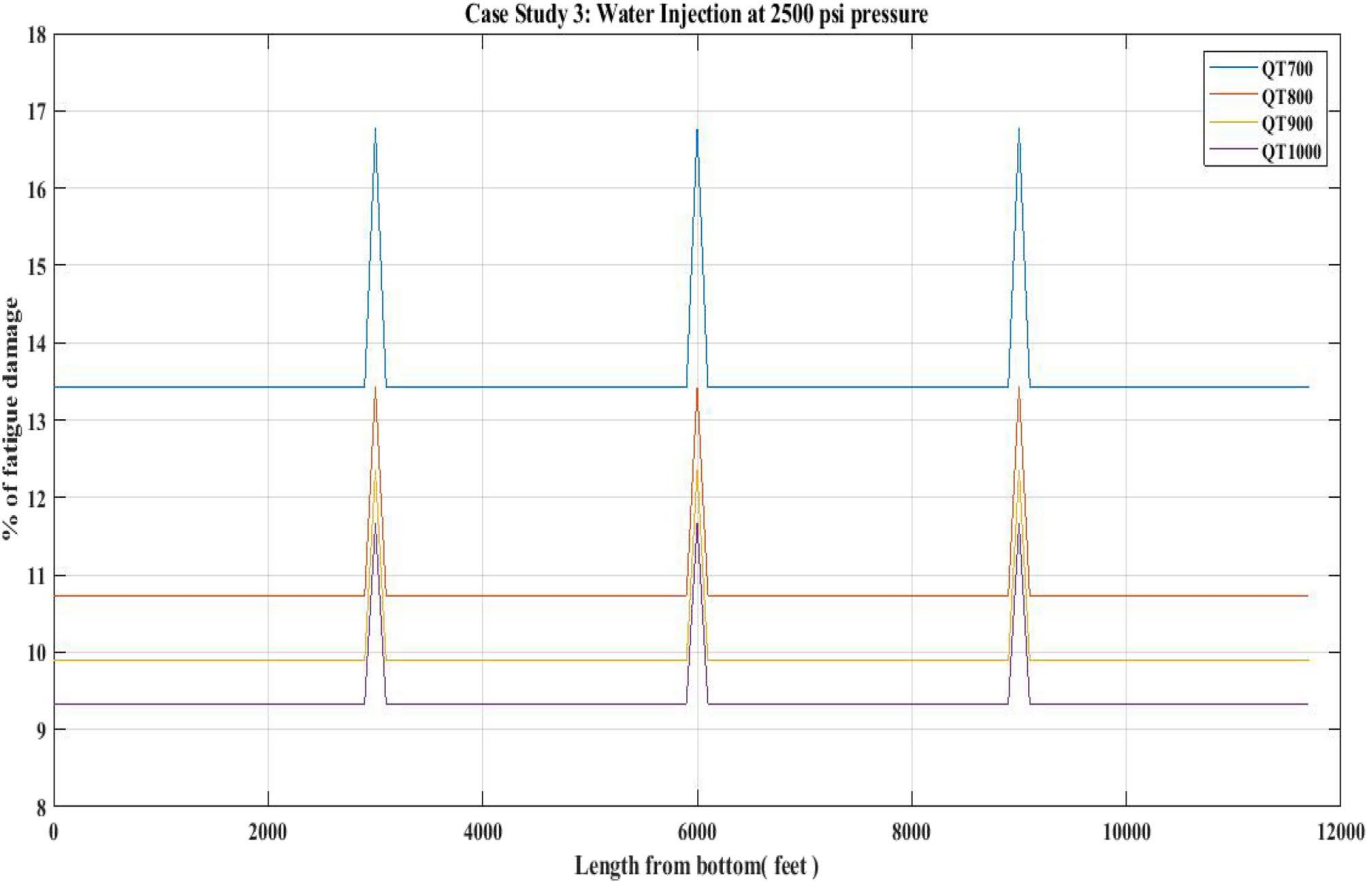
Percentage of fatigue damage with respect to water injection at 2500 psi pressure.

In the current work, fatigue damage was evaluated using MATLAB software for the CTs QT700, QT800, QT900, and QT1000. In the CTQT700 analysis, the highest fatigue damage was identified as 16.8%. The fatigue damage obtained for various CT materials is presented in Fig. 27. As seen in Fig. 27, the QT1000 material has incurred the least degree of fatigue damage. Table 13 compares the fatigue damage achieved by different CTs during the water injection activity.

#### Fatigue damage of QT 700, QT 800, QT900 and QT1000 observed for 3000 psi circulation pressure (Hot oil)

The percent fatigue damage of CTs was measured in this investigation in conjunction with a hot oil circulation operation done at 3000 psi circulation pressure. The information in Table 14 was used to forecast fatigue damage for different CT materials.

**Figure 28:**
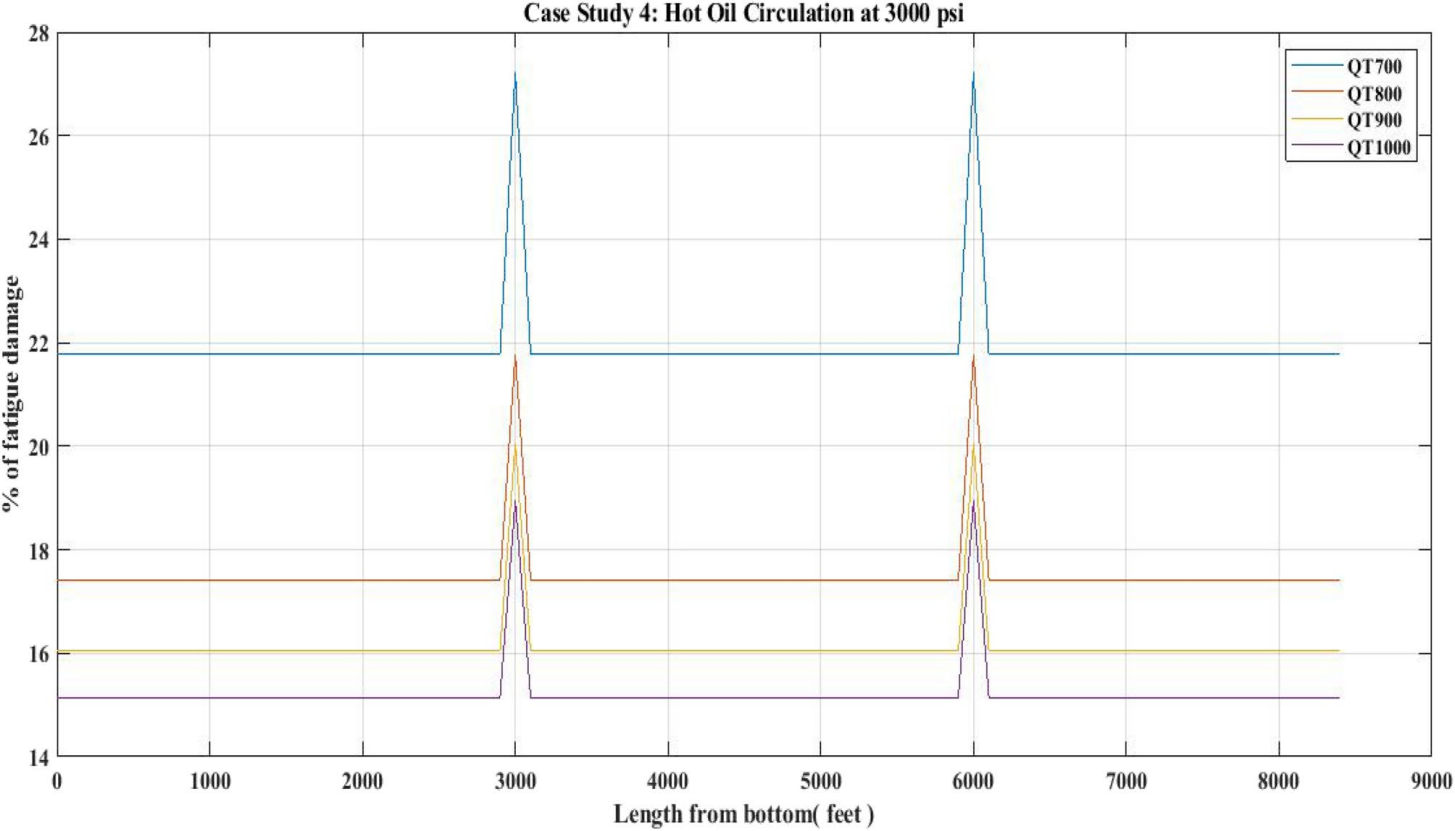
Percentage of fatigue damage with respect to hot oil circulation at 3000 psi pressure.

The current investigation observed 27% fatigue damage to the bias welded parts of the QT 700 (Fig. 28). According to Fig. 28, the QT1000 CT material suffered the least amount of fatigue damage. The increase in fatigue damage (27%) reported for the CT QT700 may be due to the high circulation pressure of 3000 psi used in the HOC work. Table 15 shows the fatigue damage for all of the CTs.

#### Fatigue damage of QT 700, QT 800, QT900 and QT1000 observed for 2000 psi nitrogen shooting job

The fatigue damage for the CTs was also investigated for a nitrogen shot job performed in an oil producing porous medium. The relevant data for the nitrogen shooting operation is shown in Table 16.

**Figure 29:**
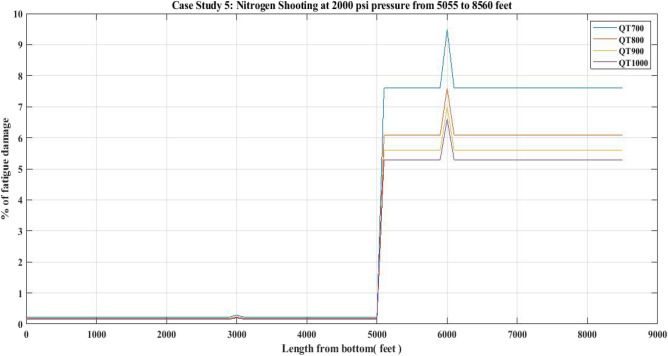
Percentage of fatigue damage with respect to Nitrogen Shooting at 2000 psi pressure.

Figure 29 displays the results of the current analysis. Table 17 shows the fatigue damage for CT observed at a circulation pressure of 2000 psi, which was used for the nitrogen shot job.

### CT elongation and temperature effect

#### For QT 800 nitrogen shooting

In this study, CT strain was investigated by using the proposed correlation for CT elongation. The current analysis was performed for a nitrogen shooting operation. Table 18 displays the field data for a well at a depth of 9000 ft. In this analysis, the BHA’s weight was considered to be zero.

**Figure 30:**
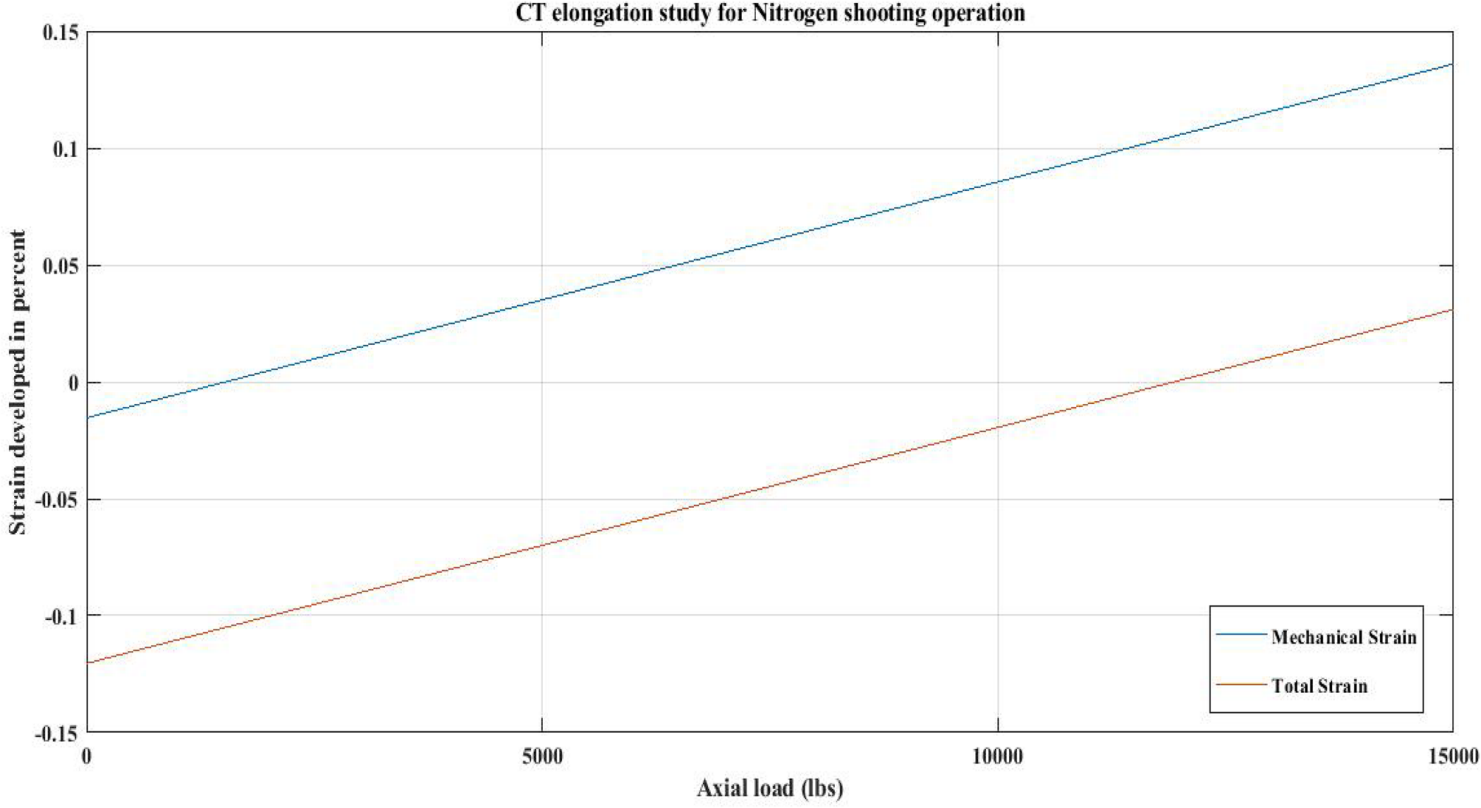
Axial load vs strain in CT (%).

The data from Table 18 was employed in the current computational analysis considering the proposed correlation (Eq. 22). The purpose of this study is to examine the mechanical strain and total strain induced on the CT as a result of the nitrogen shot operation. The results of the analysis of CT800 are shown in Fig. 30 and Table 19.

Figure 30, displays that the CT was in compression when the nitrogen circulation commenced. Furthermore, the total strain plot demonstrates that the temperature effect was significant, forcing the CT to shrink. Also, the self-weight of the pipe was insufficient to keep the CT in tension. As a result, the CT was in compression during the nitrogen pumping operation, with circulation continuing up to the axial load of 12,000 lbs (78.5 ft from the bottom). Furthermore, if the temperature effect is ignored, the mechanical strain profile in Fig. 30 reveals that the CT pipe is under compression until it reaches a 1910 lb axial load (12.5 feet from the bottom). This occurs as a result of the build-up of hoop and radial stresses in the pipe during circulation.

#### For QT 800 Hot oil circulation

The QT800 was examined in this work for a hot oil circulation operation. Table 20 offers information about the HOC job. The weight of the BHA is considered to be zero in the current analysis due to the lack of a bottom hole assembly in the well’s downhole at a depth of 10,000 feet.

**Figure 31:**
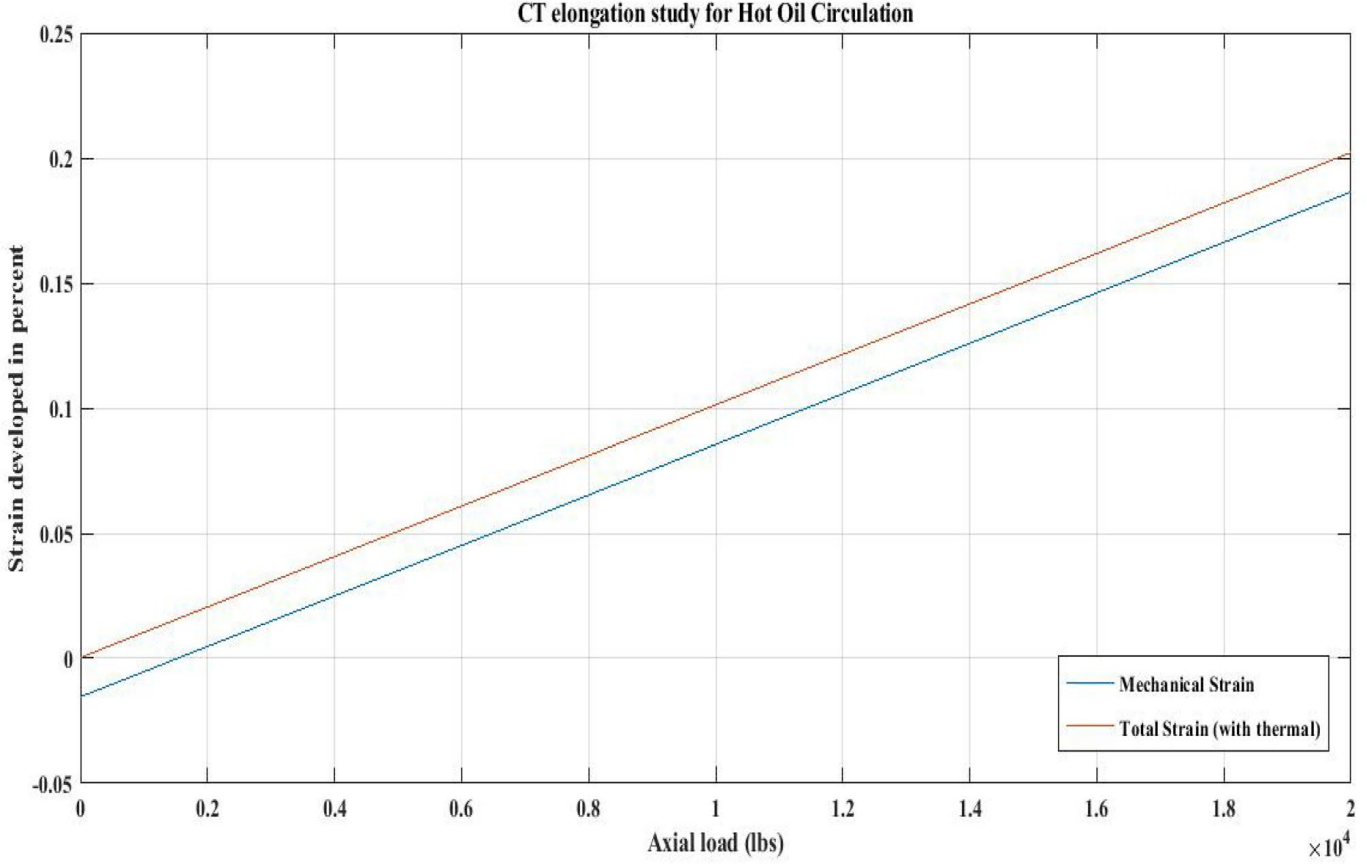
CT strain plot for a HOC job with QT800.

The relevant information from Table 20 was used to determine the mechanical and total strain applied to the CT in order to determine its elongation. The data acquired by the proposed correlation was then employed in the computational analysis to build CT strain plots. Table 21 and Fig. 31 show the comparable results for the CT strain.

The temperature strain plot in Fig. 31 shows that when hot oil is circulated, the temperature strain increases the tension in the CT along its entire length. In contrast, if the influence of temperature strain is ignored, the mechanical strain curve in Fig. 31 reveals that the CT will be in compression until an axial load of up to 1910 lbs (12.5 ft from the bottom) is achieved.

## Discussions and conclusion

### P & T limits

The results of the current study presented in Figs. 2, 3, 4, 5, 6 related to the computational assessment of the CTs (QT 800, QT 900, and QT 1000) operated in actual well conditions highlight the yield limit of the CT as well as the external pressure to which the CT can be subjected. When the external pressure was at 0 psi for QT 800, the upper limit for differential pressure (ΔP) was 11,956.67 psi in the burst region and 8555.45 psi in the collapse region (Table 4) against an axial load of 10,000 lbs. As the external pressure is increased to 2500 psi from its no external pressure condition, the ΔP burst has increased to 11,963.7 psi, and for 5000 psi, it is found to be 11,959.1 psi, both of which are higher than zero external pressure for the QT 800. When the axial load is increased to 25,000 lbs, the operating limit of QT 800 is reduced to 8367.064 psi for an external pressure of 2500 psi in the burst region, and for a 5000 psi external pressure, it is further reduced to 7565.284 psi in the burst region. However, in all cases for CT QT 800, with an increase in external pressure from 0 to 2500 psi against axial loads of 10,000 lbs and 25,000 lbs, the results of the present study in Figs. 2, 3, 4, 5, 6 display that as external pressure increases, the area of the collapse zone in the CT limit curve shrinks, making the coil tubing more prone to collapse failure than burst failure. This finding is indicative of CT being prone to collapse failure with an increase in external pressure. The findings of the current study demonstrate that the collapse pressure in different CTs increases with an increase in external pressure, which makes the CT pipe fail. This finding was also observed by different researchers, who demonstrated that during oil and gas production, external pressure causes collapse failure in the CT^15^. In addition, a CT with a lower yield strength, i.e., QT 800, would fail as break-down chances are higher under collapse pressure than QT 900 and QT 1000 CT materials (Table 5) (Figs. 1, 2, 3, 4, 5, 6). When 6% diametric growth is considered, the ΔP burst limit for QT 800 CT drops to 10,645.7 psi against an external pressure of 2500 psi, but the collapse pressure rises to (− ) 4790.12 psi from (− ) 5792.00 psi (Table 6) (Figs. 7, 8, 9, 10, 11, 12, 13, 14, 15). While examining limit curve trends, taking SF into account, CTs exhibited similar results in being prone to collapse failure (Figs. 16, 17, 18, 19, 20, 21, 22, 23, 24) (Table 17).

### Fatigue damage

 The results of the fatigue analysis examined with respect to CT operations in hot oil circulation, water injection, and nitrogen shot operations reveal that increasing internal pressure on any CT greatly increases fatigue damage. When fatigue damage was estimated for 1000 psi circulation pressure in a hot oil operation with the use of QTs 700, 800, 900, and 1000, higher fatigue damage was reported in the QT 700 CT than in the QT 800 and other CTs. In QT 700, the percentage of fatigue damage reported in the unwelded section was 1.72 and in the welded section was 2.18 (Table 9) (Fig. 25). However, when the internal pressure or circulation pressure for hot oil circulation operations was increased to 2000 psi, the fatigue percentage increased to 7.6 in the non-welded part and 9.5 in the welded part (Table 11). This trend was observed in the percentage of fatigue damage resulting from water injection as well. When the water injection circulation pressure was 2500 psi, the fatigue percentage in the then non-welded part for QT 800 was found to be 10.8 and in the welded part it was 13.3, which is lower than the fatigue observed in the case of QT 700 CT material (Table 13) (Fig. 27). Following this, the results of the nitrogen shooting operation conducted at 2000 psi reported that QT700 resulted in a higher percentage of fatigue damage for non-welded parts at 7.7 and for welded parts at 9.5 (Table 17) (Fig. 29). The above results establish that bias-welded components are more susceptible to fatigue damage than non-welded portions (Figs. 26, 27, 28, 29). Fatigue damage recorded at various portions prior to the nitrogen operation was approximately 0.2% (Fig. 29). Then, at the 3000-foot welded section from the bottom, a 0.3% surge was recorded. As seen in Fig. 29, the fatigue damage was at its lowest until the length reached 5000 feet. The fatigue damage at the welded section for the CT QT700 increased to 9.5% when the nitrogen shooting operation began at 5000 feet.

### CT elongation and temperature effect

The results of CT elongation under the influence of downhole temperature for QT 800 during nitrogen shot operation show that the total strain on the CT causes it to shrink and the CT's own weight fails to keep the CT in tension (refer to Fig. 30). Similarly, QT 800 was analysed in reference to an actual hot oil operation in upper Assam basin oil wells, and the total strain did not cause any shrinking effect, allowing CT to be in tension along its entire length (Fig. 31). Therefore, there is a disparity in the results obtained regarding the temperature effect on the CT strain observed in the cases of hot oil circulation and nitrogen shooting.

Considering the above, the current study draws the following conclusions:

i. CT pipe with a lower yield strength fails more frequently than pipe with a higher yield strength. In contrast to QT 900 and 1000 CT materials, QT 800 would degrade frequently under increasing external pressure under axial loads of 10,000 and 25,000 lbs.
ii. With increasing external pressure on the CT, greater diametric growth increases the chances of the CT failing under collapse pressure.
iii. The increase in internal pressure on CT causes a higher percentage of fatigue damage in low-yield strength CT.
iv. The welded section in CT pipe is more prone to fatigue damage than the non-welded part.
v. When total strain is considered, taking into account the temperature effect, the CT undergoes shrinking in the case of a nitrogen shot operation. However, a contrasting effect is observed when hot oil circulation is given to the same QT 800-CT material.

Finally, the current study concludes that monitoring the CT working limit in oil well intervention operations, which takes into account the effects of internal and external pressure, diametrical growth, and fatigue damage, establishes a framework for predicting when CT failure will occur. In addition, this work identifies the influence of temperature on causing CT elongation during nitrogen shooting and hot oil circulation operations. However, there is a disparity in the temperature effect on the CT strain that needs to be looked into further. On the other hand, the incorporation of this novel aspect in CT computational analysis could serve as a reference for detecting the CT service limit under varied well conditions. CT fatigue issues should be examined both qualitatively and quantitatively in order to reduce the chances of tubing failure and postpone CT’s early retirement^3,8,11^.

## Data Availability

The data used to support the findings of this study are included within the article.

## Abbreviations

*A*: Cross-sectional area of CT pipe
*CT*: Coiled tubing
*d*_*i*_: Inner diameter of CT pipe
*d*_*o*_: Outer diameter of CT pipe
*F*_*a*_: Axial load on CT pipe
*FD*: Fatigue damage
${K}_{c}$: CT operation corrosion factor
${K}_{us}$: CT ultimate strength compensation factor
${K}_{Q}$: CT life prediction reliability factor
$$\frac{\Delta L}{{L}_{i}}$$: Total strain
$$\frac{\Delta L}{{L}_{i}}\left(m\right)$$: Mechanical strain
$$\frac{\Delta L}{{L}_{i}}\left(t\right)$$: Thermal strain
*L*_*f*_: Final length of CT pipe
*L*_*i*_: Initial length of CT pipe
${N}_{1}$: No. of cycles during one tubing stroke
${N}_{m}$: CT median life
*P*: Pressure
*P*_*i*_: Internal pressure
*P*_*o*_: External pressure
*r*_*i*_: Internal radius of CT pipe
*r*_*o*_: External radius of CT pipe
*r*_*i2*_: Inside radius of the CT at maximum diameter
*r*_*o2*_: Outside radius of CT at maximum diameter
*RA*: CT area reduction, percent
${S}_{g}$: Equivalent uniaxial alternating stress on g ~ psi
${S}_{h}$: CT hoop stress due to internal pressure, psi
${S}_{m}$: CT median failure stress from tests, psi
${S}_{r}$: Equivalent uniaxial alternating stress on reel, psi
${S}_{a,g}$: CT bending stress on gooseneck, psi
${S}_{a,r}$: CT bending stress on reel, psi
*T*: Tension
*T*_*f*_: Final temperature
*T*_*i*_: Initial temperature
*w*_*2*_: Wall thickness of the CT when the CT is at maximum diameter
*W*_*BHA*_: Weight of bottom hole assembly
*µ*: Poisson’s ratio
*E*: Young’s Modulus of elasticity
*α*: Coefficient of thermal expansion, $\Delta T= {\mathrm{T}}_{\mathrm{f}}-{\mathrm{T}}_{\mathrm{i}}$
${\sigma }_{h}$: Hoop stress in CT pipe
${\sigma }_{r}$: Radial stress in CT pipe
${\sigma }_{a}$: Axial stress in CT pipe
${\sigma }_{y}$: Minimum yield stress of the CT pipe
*β*: Defined in Eq. (6)
*γ*: Defined in Eq. (6)
*δ*: Defined in Eq. (6)
*ΔP*: Difference between *P*_*i*_ and *P*_*o*_

## References

[1] Gang Zhang L, Bei Yue Q, Luo M (2017). The prediction of the low cycle fatigue life about the coiled tubing with ovality and wall thickness. J. Fail. Anal. Prev..

[2] Li L, Shen Z, Wang P (2011). Mechanism study on the coiled tubing deformation under a combination loading. Adv. Mater. Res..

[3] Shaohu L, Hui X, Feng G, Qifeng J, Jiwei W, Ting Y (2017). Coiled tubing failure analysis and ultimate bearing capacity under multi-group load. Eng. Fail. Anal..

[4] Tong S, Gao D (2018). Elastic–plastic limit load of coiled tubing under complex stress state. Arab. J. Sci. Eng..

[5] Larsen, R., Kenrick, H. A., Bell, A. Utilizing coiled tubing in mobile bay’s 22,000 TVD gas wells yields economical and technical advancements. *Soc. Pet. Eng.* SPE 38423, 10 (1997).

[6] Newman, K. R. Coiled-tubing pressure and tension limits. *Soc. Pet. Eng.* 269–278, 10.2118/23131-ms (1991).

[7] Zheng, A. Monitoring and managing coiled tubing integrity. *Oilf. Rev.* 48–56 (2015).

[8] Behenna, F. R.* et al.* Field validation of a coiled tubing fatigue model. *SPE/ICoTA coiled tubing conference exhibition 2003, CT 2003.**Soc. Pet. Eng.*10.2118/81726-ms (2018).

[9] Kale, A. V & Thorat, H. T. Control of ovality in pipe bending : A new approach. in *5th International & 26th All India Manufacturing Technology, Design and Research Conference (AIMTDR 2014) December 12th–14th, 2014 *1–5 (AIMTDR IIT Guwahati, Assam, India, 2014).

[10] Zhao C, Yu G, Chi J, Zhang J, Guo Z (2018). Theoretical and experimental investigation of coiled-tubing deformation under multiaxial cyclic loading. SPE Drill. Complet..

[11] Zhou ZM, Tan JS, Wan F, Peng B (2019). Improvement and determination of the influencing factors of coiled tubing fatigue life prediction. Adv. Mech. Eng..

[12] Li L, Shen ZX, Wang P (2013). Research the coiled tubing deformation under internal pressure and cyclic bending. Appl. Mech. Mater..

[13] Brown, P.A, & Dickerson, J. Development and use of an analytical model to predict coiled tubing diameter growth. *Soc. Pet. Eng.* SPE 38409, 1–11 (1997).

[14] Wu, J. Coiled tubing working life prediction. *Soc. Pet. Eng.* 1–7 (1995).

[15] Tong S, Gao D (2019). Elastic-plastic collapse limit analysis of coiled tubing under complex stress state. J. Pet. Sci. Eng..

[16] Liu B, Li T, Xu L, Han B, Zhuang H, Li S (2018). Effect of surface defects and internal pressure on fatigue life of coiled tubing. Adv. Eng. Res..

[17] Tipton, S. M., Smalley, E. & VanArnam D. Influence of a straightener on coiled tubing fatigue. *Coiled Tubing Well Intervention Conference Exhibition 2012*. *Soc. Pet. Eng.* (1) 216–224 10.2118/154057-ms (2012).

[18] Zhu ZL, Liang Z, Hao J (2018). Buckling load of bending coiled tubing under an injector. Adv. Mech. Eng..

[19] Padron, T., Luft, B., Kee, E. & Tipton, S. M. Fatigue life of coiled tubing with external mechanical damage.* Coiled Tubing Well Intervention Conference Exhibition 2007*. *Soc. Pet. Eng.* 379–394 10.2118/107113-ms (2007).

[20] W. P. Van Adrichem, “Coiled tubing failure statistics used to develop tubing performance indicators,” *Proc. SPE/ICoTA Coiled Tubing Roundtable Conf*, no. SPE 54478, pp. 173–182, 1999, doi: 10.2523/54478-ms.

[21] Ji Y, Zhang H, Duan Q, Cao Y (2011). Research on the analysis of coiled tubing fatigue life. Appl. Mech. Mater..

